# Prognostic value of exercise echocardiography in diabetic patients

**DOI:** 10.1186/1476-7120-7-24

**Published:** 2009-05-29

**Authors:** Joselina LM Oliveira, José AS Barreto-Filho, Carla RP Oliveira, Thaiana A Santana, Fernando D Anjos-Andrade, Érica O Alves, Adão C Nascimento-Junior, Thiago JS Góes, Nathalie O Santana, Francis L Vasconcelos, Martha A Barreto, Argemiro D'Oliveira Junior, Roberto Salvatori, Manuel H Aguiar-Oliveira, Antônio CS Sousa

**Affiliations:** 1 Department of Internal Medicine, Cardiology and Endocrinology Division, Federal University of Sergipe, Aracaju, Sergipe, Brazil; 2 Laboratory of Echocardiography of the São Lucas Hospital, Aracaju, Sergipe, Brazil; 3 Department of Internal Medicine, School of Medicine, Federal University of the Bahia, Salvador, Bahia, Brazil; 4 Division of Endocrinology, Johns Hopkins University School of Medicine, Baltimore, Maryland, USA

## Abstract

**Background:**

Coronary artery disease (CAD) is the leading cause of death in diabetic patients. Although exercise echocardiography (EE) is established as a useful method for diagnosis and stratification of risk for CAD in the general population, there are few studies on its value as a prognostic tool in diabetic patients. The purpose of this investigation was to evaluate the value of EE in predicting cardiac events in diabetics.

**Methods:**

193 diabetic patients, 97 males, 59.8 ± 9.3 yrs (mean ± SD) were submitted to EE between 2001 and 2006 and followed from 7 to 65 months with median of 29 months by phone calls and personal interviews with patients and their primary physician, and reviewing medical records and death certificates. The end points were cardiac events, defined as non-fatal myocardial infarction, late myocardial revascularization and cardiac death. Sudden death without another explanation was considered cardiac death. Survival free of end points was estimated by the Kaplan-Meier method.

**Results:**

Twenty-six cardiac events were registered in 24 individuals during the follow-up. The rates of cardiac events were 20.6 and 7% in patients with positive and negative EE, respectively (p < 0.001). Predictors of cardiac events included sedentary lifestyle, with RR of 2.57 95%CI [1.09 to 6.02] (P = 0.03) and positive EE, with RR 3.63, 95%CI [1.44 to 9.16] (P = 0.01). Patients with positive EE presented higher rates of cardiac events at 12 months (6.8% *vs*. 2.2%), p = 0.004.

**Conclusion:**

EE is a useful method to predict cardiac events in diabetic patients with suspected or known CAD.

## Background

*Diabetes mellitus *(DM) is a major risk factor for cardiovascular morbidity and mortality [1-5]. Accordingly, both the American Heart Association and American College of Cardiology define DM as an equivalent to previous coronary artery disease (CAD) for cardiovascular risk [5]. When associated with other cardiovascular risk factors, DM increases the rate of macrovascular complications.

The mean annual mortality rate of asymptomatic patients with multiple cardiovascular risk factors is 3% [6]. In diabetic patients, chronic CAD frequently presents reduced subjective symptoms, unless ventricular dysfunction is present [7]. Recognition of subclinical ischemic disease in diabetic patients and stratification of risk are important to select therapeutic interventions and to reduce the complications of cardiac events [2,7-10].

Exercise echocardiography (EE) is a valuable method for diagnosis, risk stratification and prognosis of CAD [3,11-14]. This technique has the advantages of wide availability, safety and low cost, provides information on left ventricular function, exercise capacity, and presence, location and extension of wall motion abnormalities [15]. The sensitivity of EE to detect coronary arteries obstructions varies between 70 and 100% [15,16]. EE is similar to myocardial perfusion scintigraphy in diagnosing CAD and even superior in determining its extension [15]. This is particularly relevant, as the extension of ischemia and the severity of wall motion abnormalities are considered independent and cumulative predictors of prognosis in patients with CAD [3,9].

The aim of the present study was to evaluate the prognostic value of EE in diabetic patients.

## Patients and methods

### Subjects

We retrospectively reviewed the records of 193 diabetic patients with suspected or known CAD who were referred by their physicians for diagnosis or risk stratification in the Laboratory of Echocardiography of São Lucas Hospital (city of Aracaju, State of Sergipe, Brazil) to undergo EE from March 2001 to January 2006.

All patients but one had type 2 diabetes based on referring physicians' assessment. This group was formed by mostly middle-class patients, with access to private health insurance services. Diabetic patients with significant co-morbidities, such as cancer, end-stage renal disease, severe obstructive and pulmonary disease, were not included in this analysis.

DM was defined as the presence of fasting plasma glucose ≥ 126 mg/dl on at least two occasions, or by the use of insulin or oral hypoglycemic agents [17]. Hypercholesterolemia was defined as serum total cholesterol levels ≥ 200 mg/dl (after a 12-hour fasting) or by the use of hypolipidemic agents (statins and/or fibrates). Metabolic control was assessed by the level of fasting plasma glucose, total cholesterol, LDL-cholesterol, HDL-cholesterol, and triglycerides nearest to the interview [18]. Systemic hypertension was defined as resting blood pressure measurements on upper limb ≥ 140/90 mmHg or by the use of antihypertensive medication [19].

The International Physical Activity Questionnaire (IPAQ) was used to define sedentary lifestyle. Participants were classified as active if they accumulated more than 150 minutes per week of moderate to vigorous physical activity or more than 60 minutes per week of vigorous physical activity. If not, they were considered sedentary [20]. The study was approved by the Ethics and Research Committee of Federal University of Sergipe and all subjects gave written informed consent.

### Follow-up

Information from patient follow-up was obtained by telephone interviews, household visits, or contact with the primary physician and review of medical records and/or death certificates. Cardiac events occurred after exercise echocardiography included late myocardial revascularization (LMR), nonfatal myocardial infarction (MI) – defined according to current guidelines [21] – and cardiac death, including sudden unexpected deaths without another explanation. Registered hard events were nonfatal MI and cardiac death. Deaths of presumable non-cardiac causes were all registered. The median follow-up was 29 months, range 7 to 65 months. Percentiles 25 and 75 were 16 and 43 months, respectively. Patients were divided into two groups: G1, 92 (47%) patients with EE that was positive for myocardial ischemia; G2, 101 (53%) patients with EE negative for myocardial ischemia.

### Study Protocol

#### Exercise Echocardiography

All patients underwent symptom-limited treadmill exercise testing according to the standard Bruce protocol. Heart rate (HR) was continuously recorded, and patients were strongly encouraged to reach > 85% of maximal age-predicted HR. The exercise was interrupted whenever the maximal age-predicted HR was exceeded or in the presence of the following signs and/or symptoms: chest pain, shortness of breath, muscle fatigue, hypertension (blood pressure ≥ 220/120 mmHg), hypotension (reduction of blood pressure at rest during exercise), syncope and severe arrhythmias. During the test, the individuals were continuously monitored with a three-lead electrocardiogram. Exercise test was considered positive for myocardial ischemia if there was a horizontal or down-sloping ST-segment depression, of ≥ 1 mm for men and 1.5 mm for women, at 80 ms after the J point. In the presence of electrocardiographic changes which were suggestive of the left bundle branch block, left ventricular hypertrophy, pre-excitation syndrome and use of digitalis, the ECG was considered non-diagnostic [22]. Echocardiograms were performed with Hewlett-Packard/Phillips SONOS 5500 systems, Palo Alto, Calfornia, USA. Two-dimensional echocardiographic images were obtained from the parasternal and apical windows at rest and immediately after exercise. Both, digitized and videotape-recorded or digital video display (DVD), were used for the interpretation of the studies [23]. Regional wall motion was assessed semi quantitatively by experienced echocardiographer, with level III training, as recommended by the American Society of Echocardiography. Wall motion at rest and with exercise was scored from 1 through 4 (1, normal; 2, hypokinesis; 3, akinesis; 4, dyskinesis) according to a 16-segment model [24]. Wall motion score index (WMSI), was determined at rest and peak exercise as the sum of the segmental scores divided by the number of visualized segments. In order to evaluate ischemia, wall motion abnormalities in positive EE were defined as: (a) myocardial ischemia: development of a wall motion abnormality with exercise; (b) fixed ischemia: wall motion abnormality present at rest and unchanged with exercise; (c) fixed and induced ischemia: wall motion abnormality at rest that worsens or appears in a different segment with exercise [25,26].

#### Statistical analysis

Continuous variables were reported as mean ± SD or median and interquatile range. Comparisons between groups were performed with student's *t *test. Categorical variables were summarized as percentages, and group comparisons were based on chi-square test. Survival free of events was estimated by the Kaplan-Meier method. Univariable and multivariable association of clinical, electrocardiographic and echocardiographic variables with cardiac events were assessed in the Cox proportional hazards framework and the results were summarized as risk ratios with corresponding 95% confidence intervals (CIs). Probability values less than or equal to 0.05 were considered statistically significant. Statistical analyses were performed using the SPSS 13.0 (SPSS, Chicago, IL).

## Results

### Subject characteristics

A total of 193 patients (97 men) with mean age of 59.8 ± 9.3 years (range 38 to 91 years) were studied. Fifty-two patients (26.9%) used insulin (with or without oral hypoglycemic agents), 46 patients (24%) had previous myocardial revascularization and 28 (14.5%) had previous MI. The most frequent cardiovascular risk factors in the group studied were systemic hypertension, hypercholesterolemia and sedentary lifestyle.

Clinical features of patients in groups G1 and G2 were similar, except use of insulin (34.8% vs.19.8%, P = 0.02), beta blockers (30.4% vs. 8.9%, P < 0.001) and nitrate (15.2% vs. 5.9%, P = 0.03), which were all higher in patients with positive EE. There were not differences in metabolic control in the two groups (Table 1).

**Table 1:** Clinical features of patients with abnormal (G1) and normal (G2) exercise echocardiography

VARIABLES	G1 (n = 92)	G2 (n = 101)	P
Males (%)	44 (47.8%)	53 (52.5%)	0.52
Age (Years)	60.4 ± 9.5	59.3 ± 9.1	0.42
Systemic Hypertension (%)	81 (88%)	90 (89.1%)	0.82
Fasting Plasma Glucose	143.0 ± 44.1	149.2 ± 42.6	0.83
Total Cholesterol	187.0 ± 47.8	191.6 ± 45.5	0.95
HDL-Cholesterol	50.7 ± 16.8	48.3 ± 13.0	0.55
LDL-Cholesterol	109.5 ± 46.8	110.1 ± 40.3	0.7
Triglycerides	163.2 ± 142.8	173.9 ± 83.2	0.43
Cigarette Smoking (%)	22 (23.9%)	19 (18.8%)	0.39
Sedentary (%)	45 (48.9%)	45 (44.6%)	0.54
BMI	27.9 ± 4.2	28.9 ± 3.8	0.11
Family History of IHD (%)	38 (41.3%)	34 (33.7%)	0.27
Alcoholism (%)	40 (43.5%)	45 (44.6%)	0.88
Insulin Therapy (%)	32 (34.8%)	20 (19.8%)	0.02
Oral Hypoglycemic Agents (%)	68 (73.9%)	83 (82.2%)	0.16
Beta Blocker Therapy (%)	43 (47.8%)	29 (29.3%)	0.009
Nitrates Therapy (%)	33 (36.7%)	6 (6.1%)	<0.001
Ca^++^Channel Blocker Therapy (%)	33 (36.7%)	30 (30.3%)	0.35

BMI = Body Mass Index

IHD = Ischemic heart disease

### Follow-up

Twenty-four patients (12.4%) presented cardiac events during the follow-up. Group G1 had 19 cardiac events: 4 MI, 11 LMR and 4 cardiac deaths. Seven cardiac events occurred in Group G2: 2 MI, 3 LMR and 2 cardiac deaths. One patient in each group had two events during the follow-up (MI and LMR) and the overall rate of these major cardiac events in both groups was 13.5% LMR was more frequent in group G1 (12% vs. 3%; p < 0.001) (Table 2). The analysis of cardiac events in diabetics with normal EE demonstrated that MI's occurred 10 and 25 months after the echocardiography, while LMR's occurred after periods of 25, 21 and 12 months. The cardiac deaths happened 27 and 53 months after the EE.

**Table 2:** Hemodynamic and echocardiographic features of patients with abnormal (G1) and normal (G2) exercise echocardiography

VARIABLES	G1 (n = 92)	G2 (n = 101)	P
Failure to Achieve 85% of the Maximal Age-predicted Heart Rate	30 (32.6%)	31 (30.7%)	0.77
Achieved 85% of the Maximal Age-predicted Heart Rate	32 (34.8%)	24 (23.8%)	0.09
Achieved Maximal Age-predicted Heart Rate	12 (13%)	23 (22.8%)	0.08
Achieved Above Maximal Age-predicted Heart Rate	3 (7.5%)	16 (32.7%)	0.004
Resting Heart Rate	113.5 ± 35.9	116.2 ± 39.3	0.62
Peak Exercise Heart Rate	164 ± 29.2	170.5 ± 25.6	0.11
Resting Systolic Blood Pressure	159.6 ± 30.4	163.8 ± 36.3	0.38
Resting Diastolic Blood Pressure	86.7 ± 18.6	83.1 ± 14.7	0.14
Peak Exercise Systolic Blood Pressure	162.2 ± 32.9	163 ± 31.2	0.86
Peak Exercise Diastolic Blood Pressure	88.4 ± 11.6	87.5 ± 9.8	0.56
Final Systolic Blood Pressure	107.6 ± 35.2	108.8 ± 36	0.81
Final Diastolic Blood Pressure	35.8 ± 40.3	38.1 ± 38.6	0.68
Resting Ejection Fraction	0.64 ± 0.07	0.66 ± 0.05	0.008
WMSI* at Rest	1.06 (0.12)^†^	1.00 (0.00)^†^	< 0.001
WMSI* Peak Exercise	1.12 (0.18)^†^	1.00 (0.00)^†^	< 0.001

Values are means ± SD

WMSI = Wall Motion Score Index

^† ^Median (interquatile range) Mann-Whitney test

### Exercise echocardiography

Diabetic patients that achieved above the maximal age-predicted heart rate during EE presented positive EE less frequently that the ones that did not achieved it (7.5% vs. 32.7%, P = 0.004).

There was no difference in resting and peak exercise heart rates and blood pressure between G1 and G2. Resting ejection fraction was higher in group G2 (0.64 ± 0.07 vs. 0.66 ± 0.05, P = 0.01). Patients from group G1 presented higher WMSI at rest and peak exercise (P < 0.001) (Table 3).

**Table 3:** Cardiac events in patients with abnormal (G1) and normal (G2) exercise echocardiography

VARIABLES	G1 (n = 92)	G2 (n = 101)	P
Myocardial infarction	4 (4.3%)	2 (2%)	0.113
Late myocardial reperfusion	11 (12%)	3 (3%)	<0.001
Death	4 (4.3%)	2 (2%)	0.113
Total	19 (20.6%)	7 (7%)	<0.001

Independent predictors of cardiac events included sedentary lifestyle, with RR of 2.57 95%CI [1.09 to 6.02] (P = 0.03) and positive EE, with RR 3.63, 95%CI [1.44 to 9.16] (P = 0.01).

**Figure 1:**
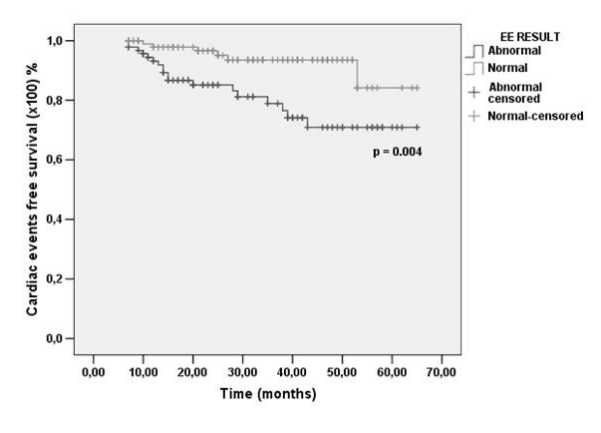
**Kaplan-Meier curves for survival free of cardiac events in patients with normal (gray line) *vs*. abnormal (black line) exercise echocardiography (EE)**.

In the analysis of survival free of end points, the events rate was higher in patients with ischemic EE, in comparison to those with normal result at 12 (6.8% vs. 2.2%), 24 (15% vs. 3.4%) and 65 months (29% vs. 15.8%), P = 0.004 (Fig. 1).

**Figure 2:**
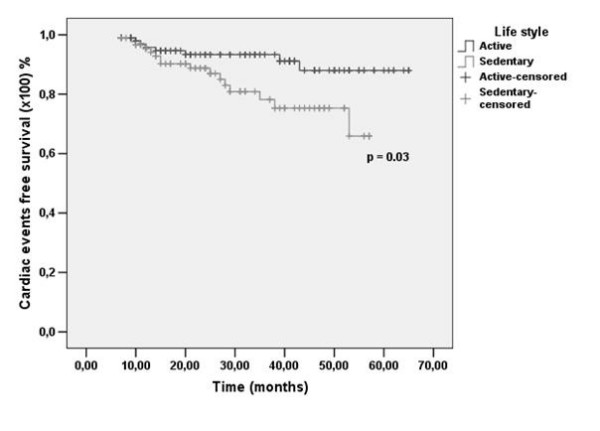
**Kaplan-Meier curves for survival free of cardiac events in diabetic patients according to lifestyle**. Black line: active patients; Gray line: sedentary patients.

Sedentary diabetic patients had higher rates of cardiac events in comparison to active diabetics at 12 (4.6% vs. 4.2%), 24 (11.3% vs. 6.7%) and 57 months (34.1% vs. 12%), P = 0.03 (Fig. 2)(Additional Files 1, 2, 3, 4 and 5).

## Discussion

Identification of patients with subclinical disease and high risk of future cardiac events occurrence is a strategy that aims to reduce the risk of complications of CAD [2,8]. The value of EE in prognostic stratification of diabetic patients with known or suspected CAD has been previously reported [3,27].

In the present study, the number of positive EE was higher than reported in the general population [14], confirming the higher risk of CAD in diabetic patients. A higher number of patients with normal EE reached above maximal age-predicted heart rate (P = 0.004). Patients with ischemic EE presented higher levels of WMSI at rest and during peak of exercise (P < 0.0001). Resting ejection fraction was lower in diabetics with positive EE (0.64 ± 0.07 vs. 0.66 ± 0.05, P = 0.01), although both fell within normal values, suggesting that the G1 group did not have heart failure.

The rate of cardiac events was higher in patients with ischemic EE in comparison to those with a normal result, despite comparable metabolic control. In a similar study, Elhendy et al. [3] followed up 563 diabetic patients with known or suspected ischemic heart disease who underwent EE for a median period of three years. Fifty patients (9%) experienced cardiac events during the follow-up. Similar to our study, the rate of events was higher in patients with positive EE, in comparison to patients with normal results (11.9% *vs*. 1.8% in three years). The frequency of hard events was higher than ours, possibly related to the socio-economic profile of our patients, with access to private health insurance services and high quality of medical care. Alternatively, the high number of LMR may have reduced the number of hard events in our group. In their study, none of the patients with normal EE presented cardiac events in the first two years of follow up. However, the rate of events increased gradually after two years, achieving 7.6% by the end of the fifth year. Our patients behaved similarly, since in patients with normal EE only one event occurred during the first year of follow-up, and the rate of events increased gradually after the second year and reached 15.8% by the end of the fifth year. The authors related this finding to progression of ischemic disease and recommended repeating EE after two years, as a strategy for reassessing the risk status of patients with an initial normal result. Our data suggest that it is better to repeat the EE each year, because one MI occurred 10 months and one LMR 12 months after a normal EE. We also found by multivariate analysis that sedentary lifestyle and positive EE were predictors for cardiac events, while to Elhendy et al., MI, ejection fraction at rest, and percentage of ischemic segments during exercise were the most important predictors [3].

Garrido et al. [11] assessed the value of EE for prediction of cardiac events in 214 patients with DM. Twenty-eight developed cardiac events (15 cardiac deaths and 13 MI) during a follow-up of 44 ± 16 months. This study described the following independent risk factors as predictor of future cardiac events: use of insulin, ventricular ejection fraction in peak exercise and, similar to our study, myocardial ischemia detected by EE. More recently, Cortigiani et al. [28] studied the prognostic value of pharmacological stress echocardiography in 149 diabetic and 786 non-diabetic patients with chest pain and intermediate to high threshold positive exercise results, with a median follow-up of 26 months. They recorded 51 deaths, 29 MI and 79 LMR, with a rate of major events of 26% in diabetics, higher than our global rate of 13.5%, probably due to more severe cardiovascular disease in the entry of that study. They concluded that a non-ischemic test predicts a 6-month period free of major events, and a 2% major event rate at 1-year-follow-up in both populations, with marked increase of major events rate in diabetic patients afterwards. These data are in agreement with our finding of a gradual increase in risk after the second year and a 15.8% rate by the end of the fifth year.

Sozzi et al demonstrated that an abnormal dobutamine stress echocardiography was associated with a higher mortality compared with a normal dobutamine stress echocardiogram (p = 0.03) in asymptomatic diabetic patients with no previous CAD [27].

The last consensus of stress echocardiography experts [29] stated that inducible myocardial ischemia in echocardiography by physical or pharmacological stress present a comparable prognostic value. It has also been suggested that patients with ischemic left ventricular dysfunction and a significant amount of viable myocardium have better prognosis, with lower perioperative mortality, greater improvements in global and regional left ventricular function and higher long-term survival after revascularization than patients with non-viable myocardium. In addition to that, coronary flow reserve and wall motion analysis offer complementary data during stress echocardiography. The combination of these two parameters improves the prognostic value. A reduced coronary flow reserve is a parameter of ischemic severity in risk stratification of EE response whereas patients with a negative test for wall motion criteria and normal coronary flow reserve have a favorable outcome during dypiridamole stress echocardiography.

At this moment, our echocardiography laboratory has a larger data base than the one used in this manuscript, and a research is currently being developed concerning prognostic value of EE in all the patients, diabetic and non-diabetic. Therefore, in this study, we cannot assess the impact of revascularization procedures in both groups. Previous reports demonstrated that diabetic patients with a normal EE result present worse outcomes in comparison to their age-matched non-diabetic counterparts [30]. In addition to that, Cortigiani et al found abnormal coronary flow reserve in the left anterior descending to be a strong, independent and additive prognostic indicator in a large cohort of diabetic and non-diabetic patients with known or suspected CAD and negative dypiridamole stress echocardiography. Thus, a negative test was less prognostically benign in diabetic patients than in age-matched non-diabetic patients [31].

Our study has some important limitations. One is the lack of data on the duration of DM. Second, the group of diabetics studied was formed by middle-class patients, with access to private health insurance services. Therefore, the results of this study may not be valid to populations of different socio-economic backgrounds. Third, post-test bias could not be eliminated, since the EE results were available to the treating physicians. Therefore, a test result positive for myocardial ischemia may have influenced the decision of performing myocardial revascularization, so that patients with a higher risk may have undergone revascularization, possibly reducing the rate of hard events. In addition, positive results in EE may have influenced in the choice of medication for these patients, also reducing the possibility of occurrence of cardiac events. These interventions, however, would reduce rather than increase the risk of ischemic events. Finally, we cannot rule out that a subset of subjects that we could not locate may have died. However, the prevalence of normal and abnormal EE in this group was almost identical, making it unlikely that this factor may have altered significant our findings.

## Conclusion

EE is a useful method to define survival free of cardiac events in diabetic patients with known or suspected ischemic heart disease, providing additional prognostic information to clinical and exercise electrocardiographic variables at rest. This technique may be useful for prognostic evaluation and risk stratification of patients who have DM.

## Abbreviations

BMI: body mass index; CAD: coronary artery disease; DM: diabetes mellitus; DVD: digital video display; ECG: electrocardiogram; EE: exercise echocardiography; HR: heart rate; MI: myocardial infarction; LMR: late myocardial revascularization; WMSI: wall motion score index.

## Competing interests

The authors declare that they have no competing interests.

## Authors' contributions

All authors contributed to this work, read and approved the final manuscript.

## Supplementary Material

Additional file 1 **RS, female patient, 73 years-old, active, BMI = 21 kg/m^2^, complaining of typical chest pain, hypertensive, with family history of coronary artery disease and previous exercise  testing negative for myocardial ischemia**. In exercise echocardiography, left ventricular mass index = 106.1 g/m^2^. Ejection fraction = 0.69, peak exercise heart rate = 145 beats/min, WMSI in peak exercise = 1.13. Time spent in treadmill exercise = 6.57 minutes, 2.5 mph, achieved second stage in Bruce protocol. In peak exercise, presented hypokinesis in anterior and lateral-apical walls. Click here for file

Additional file 2 **ILA,female patient, 61 years-old, active, BMI = 29.9 kg/m^2^, complaining of atypical chest pain, hypertensive, dyslipidemic, with family history of coronary artery disease and previous exercise testing negative for myocardial ischemia**. In exercise echocardiography, left ventricular mass index = 88.5 g/m^2^, ejection fraction = 0.69, peak exercise heart rate = 150 beats/min, WMSI in peak exercise = 1.13. time spent in treadmill exercise = 9 minutes, 3.4 mph, achieved the third stage of Bruce protocol. In peak exercise, presented hypokinesis in anterior and lateral-apical walls. Click here for file

Additional file 3 **MDA, female patient, 47 years-old, sedentary lifestyle, BMI = 32 kg/m^2^, complaining of atypical chest pain, hypertensive, previous exercise testing positive for myocardial ischemia**. In exercise echocardiography, presented left ventricular mass index = 79.8 g/m^2^, ejection fraction = 0.67, peak exercise heart rate = 174 beats/min, WMSI in peak exercise = 1. Time spent in treadmill exercise = 8.32 minutes, 2.5 mph, achieved the second stage in Bruce protocol. Wall motion in peak exercise was normal. Click here for file

Additional file 4 **JCA male patient, 43 years-old, active, BMI = 22.46 kg/m^2^**. History of myocardial infarction three years ago, complaining of atypical chest pain, hypertensive, dyslipidemic, with family history of coronary artery disease and precious exercise testing negative for myocardial ischemia. In exercise echocardiography, presented left ventricular mass index = 114.8 g/m^2^, ejection fraction = 0.56, peak exercise heart rate = 183 beats/min, WMSI in peak exercise = 1.13. Time spent in treadmill exercise = 12.53 minutes, 5.0 mph, achieved the fifth stage in Bruce protocol. Both at rest and in peak exercise presented hypokinesis in inferior-basal and inferior-medial walls. Click here for file

Additional file 5 **Output of statistical analyses performed in SPSS 13.0**. Click here for file
