# Supplementary material for: Prognostic value of exercise echocardiography in diabetic patients
Source: Cardiovasc Ultrasound. 2009 May 29;7:24. doi: 10.1186/1476-7120-7-24 (PMC2700081; doi:10.1186/1476-7120-7-24)
Supplement: Additional file 5 — Output of statistical analyses performed in SPSS 13.0. [file 1476-7120-7-24-S5.doc]

| DIABETES MELLITUS | | | | | |
| --- | --- | --- | --- | --- | --- |
|  |  | **Frequency** | **Percent** | **Valid Percent** | **Cumulative Percent** |
| **Valid** | **true** | 193 | 100.0 | 100.0 | 100.0 |

| EE RESULT | | | | | |
| --- | --- | --- | --- | --- | --- |
|  |  | **Frequency** | **Percent** | **Valid Percent** | **Cumulative Percent** |
| **Valid** | **Ischemic** | 92 | 47.7 | 47.7 | 47.7 |
|  | **Normal** | 101 | 52.3 | 52.3 | 100.0 |
|  | **Total** | 193 | 100.0 | 100.0 |  |

| Percentiles | | | | | | | | |
| --- | --- | --- | --- | --- | --- | --- | --- | --- |
|  |  | **Percentiles** | | | | | | |
|  |  | **5** | **10** | **25** | **50** | **75** | **90** | **95** |
| **Weighted Average(Definition 1)** | **Time past until the event after EE** | 7.7000 | 10.0000 | **16.0** | **29.0** | **43.0** | **54.2** | 57.3000 |
| **Tukey's Hinges** | **Time past until the event after EE** |  |  | 16.0000 | 29.0000 | 43.0000 |  |  |

**Kaplan-Meier**

| Case Processing Summary | | | | |
| --- | --- | --- | --- | --- |
| **EE normal/ischemic** | **Total N** | **N of Events** | **Censored** | |
|  |  |  | **N** | **Percent** |
| **G1 – Ischemic** | 92 | 18 | 74 | 80.4% |
| **G2 – Normal** | 101 | 6 | 95 | 94.1% |
| **Overall** | 193 | 24 | 169 | 87.6% |

| Survival Table | | | | | | | |
| --- | --- | --- | --- | --- | --- | --- | --- |
| **EE normal/ischemic** |  | **Time** | **Status** | **Cumulative Proportion Surviving at the Time** | | **N of Cumulative Events** | **N of Remaining Cases** |
|  |  |  |  | **Estimate** | **Std. Error** |  |  |
| **G1 - Ischemic** | **1** | 7.000 | WITH CARDIAC EVENT | . | . | 1 | 91 |
|  | **2** | 7.000 | WITH CARDIAC EVENT | .978 | .015 | 2 | 90 |
|  | **3** | 7.000 | WITHOUT CARDIAC EVENT | . | . | 2 | 89 |
|  | **4** | 7.000 | WITHOUT CARDIAC EVENT | . | . | 2 | 88 |
|  | **5** | 9.000 | WITH CARDIAC EVENT | .967 | .019 | 3 | 87 |
|  | **6** | 9.000 | WITHOUT CARDIAC EVENT | . | . | 3 | 86 |
|  | **7** | 10.000 | WITH CARDIAC EVENT | .956 | .022 | 4 | 85 |
|  | **8** | 10.000 | WITHOUT CARDIAC EVENT | . | . | 4 | 84 |
|  | **9** | 10.000 | WITHOUT CARDIAC EVENT | . | . | 4 | 83 |
|  | **10** | 10.000 | WITHOUT CARDIAC EVENT | . | . | 4 | 82 |
|  | **11** | 10.000 | WITHOUT CARDIAC EVENT | . | . | 4 | 81 |
|  | **12** | 11.000 | WITH CARDIAC EVENT | .944 | .024 | 5 | 80 |
|  | **13** | 11.000 | WITHOUT CARDIAC EVENT | . | . | 5 | 79 |
|  | **14** | 11.000 | WITHOUT CARDIAC EVENT | . | . | 5 | 78 |
|  | **15** | 11.000 | WITHOUT CARDIAC EVENT | . | . | 5 | 77 |
|  | **16** | 11.000 | WITHOUT CARDIAC EVENT | . | . | 5 | 76 |
|  | **17** | 12.000 | WITH CARDIAC EVENT | .932 | .027 | 6 | 75 |
|  | **18** | 12.000 | WITHOUT CARDIAC EVENT | . | . | 6 | 74 |
|  | **19** | 12.000 | WITHOUT CARDIAC EVENT | . | . | 6 | 73 |
|  | **20** | 13.000 | WITH CARDIAC EVENT | .919 | .029 | 7 | 72 |
|  | **21** | 14.000 | WITH CARDIAC EVENT | . | . | 8 | 71 |
|  | **22** | 14.000 | WITH CARDIAC EVENT | .893 | .034 | 9 | 70 |
|  | **23** | 14.000 | WITHOUT CARDIAC EVENT | . | . | 9 | 69 |
|  | **24** | 14.000 | WITHOUT CARDIAC EVENT | . | . | 9 | 68 |
|  | **25** | 15.000 | WITH CARDIAC EVENT | . | . | 10 | 67 |
|  | **26** | 15.000 | WITH CARDIAC EVENT | .867 | .038 | 11 | 66 |
|  | **27** | 15.000 | WITHOUT CARDIAC EVENT | . | . | 11 | 65 |
|  | **28** | 15.000 | WITHOUT CARDIAC EVENT | . | . | 11 | 64 |
|  | **29** | 15.000 | WITHOUT CARDIAC EVENT | . | . | 11 | 63 |
|  | **30** | 16.000 | WITHOUT CARDIAC EVENT | . | . | 11 | 62 |
|  | **31** | 17.000 | WITHOUT CARDIAC EVENT | . | . | 11 | 61 |
|  | **32** | 17.000 | WITHOUT CARDIAC EVENT | . | . | 11 | 60 |
|  | **33** | 18.000 | WITHOUT CARDIAC EVENT | . | . | 11 | 59 |
|  | **34** | 18.000 | WITHOUT CARDIAC EVENT | . | . | 11 | 58 |
|  | **35** | 19.000 | WITHOUT CARDIAC EVENT | . | . | 11 | 57 |
|  | **36** | 19.000 | WITHOUT CARDIAC EVENT | . | . | 11 | 56 |
|  | **37** | 19.000 | WITHOUT CARDIAC EVENT | . | . | 11 | 55 |
|  | **38** | 20.000 | WITH CARDIAC EVENT | .851 | .040 | 12 | 54 |
|  | **39** | 20.000 | WITHOUT CARDIAC EVENT | . | . | 12 | 53 |
|  | **40** | 20.000 | WITHOUT CARDIAC EVENT | . | . | 12 | 52 |
|  | **41** | 20.000 | WITHOUT CARDIAC EVENT | . | . | 12 | 51 |
|  | **42** | 20.000 | WITHOUT CARDIAC EVENT | . | . | 12 | 50 |
|  | **43** | 22.000 | WITHOUT CARDIAC EVENT | . | . | 12 | 49 |
|  | **44** | 22.000 | WITHOUT CARDIAC EVENT | . | . | 12 | 48 |
|  | **45** | 23.000 | WITHOUT CARDIAC EVENT | . | . | 12 | 47 |
|  | **46** | 24.000 | WITHOUT CARDIAC EVENT | . | . | 12 | 46 |
|  | **47** | 24.000 | WITHOUT CARDIAC EVENT | . | . | 12 | 45 |
|  | **48** | 25.000 | WITHOUT CARDIAC EVENT | . | . | 12 | 44 |
|  | **49** | 25.000 | WITHOUT CARDIAC EVENT | . | . | 12 | 43 |
|  | **50** | 28.000 | WITH CARDIAC EVENT | .832 | .044 | 13 | 42 |
|  | **51** | 29.000 | WITH CARDIAC EVENT | .812 | .047 | 14 | 41 |
|  | **52** | 29.000 | WITHOUT CARDIAC EVENT | . | . | 14 | 40 |
|  | **53** | 29.000 | WITHOUT CARDIAC EVENT | . | . | 14 | 39 |
|  | **54** | 31.000 | WITHOUT CARDIAC EVENT | . | . | 14 | 38 |
|  | **55** | 32.000 | WITHOUT CARDIAC EVENT | . | . | 14 | 37 |
|  | **56** | 32.000 | WITHOUT CARDIAC EVENT | . | . | 14 | 36 |
|  | **57** | 35.000 | WITH CARDIAC EVENT | .789 | .051 | 15 | 35 |
|  | **58** | 35.000 | WITHOUT CARDIAC EVENT | . | . | 15 | 34 |
|  | **59** | 37.000 | WITHOUT CARDIAC EVENT | . | . | 15 | 33 |
|  | **60** | 38.000 | WITH CARDIAC EVENT | .765 | .055 | 16 | 32 |
|  | **61** | 39.000 | WITH CARDIAC EVENT | .741 | .058 | 17 | 31 |
|  | **62** | 39.000 | WITHOUT CARDIAC EVENT | . | . | 17 | 30 |
|  | **63** | 39.000 | WITHOUT CARDIAC EVENT | . | . | 17 | 29 |
|  | **64** | 40.000 | WITHOUT CARDIAC EVENT | . | . | 17 | 28 |
|  | **65** | 40.000 | WITHOUT CARDIAC EVENT | . | . | 17 | 27 |
|  | **66** | 41.000 | WITHOUT CARDIAC EVENT | . | . | 17 | 26 |
|  | **67** | 42.000 | WITHOUT CARDIAC EVENT | . | . | 17 | 25 |
|  | **68** | 42.000 | WITHOUT CARDIAC EVENT | . | . | 17 | 24 |
|  | **69** | 42.000 | WITHOUT CARDIAC EVENT | . | . | 17 | 23 |
|  | **70** | 43.000 | WITH CARDIAC EVENT | .709 | .064 | 18 | 22 |
|  | **71** | 43.000 | WITHOUT CARDIAC EVENT | . | . | 18 | 21 |
|  | **72** | 46.000 | WITHOUT CARDIAC EVENT | . | . | 18 | 20 |
|  | **73** | 47.000 | WITHOUT CARDIAC EVENT | . | . | 18 | 19 |
|  | **74** | 48.000 | WITHOUT CARDIAC EVENT | . | . | 18 | 18 |
|  | **75** | 49.000 | WITHOUT CARDIAC EVENT | . | . | 18 | 17 |
|  | **76** | 50.000 | WITHOUT CARDIAC EVENT | . | . | 18 | 16 |
|  | **77** | 50.000 | WITHOUT CARDIAC EVENT | . | . | 18 | 15 |
|  | **78** | 52.000 | WITHOUT CARDIAC EVENT | . | . | 18 | 14 |
|  | **79** | 53.000 | WITHOUT CARDIAC EVENT | . | . | 18 | 13 |
|  | **80** | 55.000 | WITHOUT CARDIAC EVENT | . | . | 18 | 12 |
|  | **81** | 56.000 | WITHOUT CARDIAC EVENT | . | . | 18 | 11 |
|  | **82** | 56.000 | WITHOUT CARDIAC EVENT | . | . | 18 | 10 |
|  | **83** | 56.000 | WITHOUT CARDIAC EVENT | . | . | 18 | 9 |
|  | **84** | 57.000 | WITHOUT CARDIAC EVENT | . | . | 18 | 8 |
|  | **85** | 57.000 | WITHOUT CARDIAC EVENT | . | . | 18 | 7 |
|  | **86** | 57.000 | WITHOUT CARDIAC EVENT | . | . | 18 | 6 |
|  | **87** | 58.000 | WITHOUT CARDIAC EVENT | . | . | 18 | 5 |
|  | **88** | 58.000 | WITHOUT CARDIAC EVENT | . | . | 18 | 4 |
|  | **89** | 60.000 | WITHOUT CARDIAC EVENT | . | . | 18 | 3 |
|  | **90** | 61.000 | WITHOUT CARDIAC EVENT | . | . | 18 | 2 |
|  | **91** | 62.000 | WITHOUT CARDIAC EVENT | . | . | 18 | 1 |
|  | **92** | 65.000 | WITHOUT CARDIAC EVENT | . | . | 18 | 0 |
| **G2 - Normal** | **1** | 7.000 | WITHOUT CARDIAC EVENT | . | . | 0 | 100 |
|  | **2** | 7.000 | WITHOUT CARDIAC EVENT | . | . | 0 | 99 |
|  | **3** | 7.000 | WITHOUT CARDIAC EVENT | . | . | 0 | 98 |
|  | **4** | 7.000 | WITHOUT CARDIAC EVENT | . | . | 0 | 97 |
|  | **5** | 7.000 | WITHOUT CARDIAC EVENT | . | . | 0 | 96 |
|  | **6** | 8.000 | WITHOUT CARDIAC EVENT | . | . | 0 | 95 |
|  | **7** | 9.000 | WITHOUT CARDIAC EVENT | . | . | 0 | 94 |
|  | **8** | 9.000 | WITHOUT CARDIAC EVENT | . | . | 0 | 93 |
|  | **9** | 9.000 | WITHOUT CARDIAC EVENT | . | . | 0 | 92 |
|  | **10** | 10.000 | WITH CARDIAC EVENT | .989 | .011 | 1 | 91 |
|  | **11** | 12.000 | WITHOUT CARDIAC EVENT | .978 | .015 | 2 | 90 |
|  | **12** | 12.000 | WITHOUT CARDIAC EVENT | . | . | 2 | 89 |
|  | **13** | 12.000 | WITHOUT CARDIAC EVENT | . | . | 2 | 88 |
|  | **14** | 13.000 | WITHOUT CARDIAC EVENT | . | . | 2 | 87 |
|  | **15** | 14.000 | WITHOUT CARDIAC EVENT | . | . | 2 | 86 |
|  | **16** | 15.000 | WITHOUT CARDIAC EVENT | . | . | 2 | 85 |
|  | **17** | 16.000 | WITHOUT CARDIAC EVENT | . | . | 2 | 84 |
|  | **18** | 16.000 | WITHOUT CARDIAC EVENT | . | . | 2 | 83 |
|  | **19** | 16.000 | WITHOUT CARDIAC EVENT | . | . | 2 | 82 |
|  | **20** | 17.000 | WITHOUT CARDIAC EVENT | . | . | 2 | 81 |
|  | **21** | 18.000 | WITHOUT CARDIAC EVENT | . | . | 2 | 80 |
|  | **22** | 20.000 | WITHOUT CARDIAC EVENT | . | . | 2 | 79 |
|  | **23** | 21.000 | WITH CARDIAC EVENT | .966 | .019 | 3 | 78 |
|  | **24** | 21.000 | WITHOUT CARDIAC EVENT | . | . | 3 | 77 |
|  | **25** | 21.000 | WITHOUT CARDIAC EVENT | . | . | 3 | 76 |
|  | **26** | 21.000 | WITHOUT CARDIAC EVENT | . | . | 3 | 75 |
|  | **27** | 21.000 | WITHOUT CARDIAC EVENT | . | . | 3 | 74 |
|  | **28** | 22.000 | WITHOUT CARDIAC EVENT | . | . | 3 | 73 |
|  | **29** | 23.000 | WITHOUT CARDIAC EVENT | . | . | 3 | 72 |
|  | **30** | 23.000 | WITHOUT CARDIAC EVENT | . | . | 3 | 71 |
|  | **31** | 24.000 | WITHOUT CARDIAC EVENT | . | . | 3 | 70 |
|  | **32** | 24.000 | WITHOUT CARDIAC EVENT | . | . | 3 | 69 |
|  | **33** | 24.000 | WITHOUT CARDIAC EVENT | . | . | 3 | 68 |
|  | **34** | 24.000 | WITHOUT CARDIAC EVENT | . | . | 3 | 67 |
|  | **35** | 24.000 | WITHOUT CARDIAC EVENT | . | . | 3 | 66 |
|  | **36** | 25.000 | WITH CARDIAC EVENT | .951 | .024 | 4 | 65 |
|  | **37** | 25.000 | WITHOUT CARDIAC EVENT | . | . | 4 | 64 |
|  | **38** | 25.000 | WITHOUT CARDIAC EVENT | . | . | 4 | 63 |
|  | **39** | 25.000 | WITHOUT CARDIAC EVENT | . | . | 4 | 62 |
|  | **40** | 25.000 | WITHOUT CARDIAC EVENT | . | . | 4 | 61 |
|  | **41** | 26.000 | WITHOUT CARDIAC EVENT | . | . | 4 | 60 |
|  | **42** | 27.000 | WITH CARDIAC EVENT | .935 | .028 | 5 | 59 |
|  | **43** | 27.000 | WITHOUT CARDIAC EVENT | . | . | 5 | 58 |
|  | **44** | 27.000 | WITHOUT CARDIAC EVENT | . | . | 5 | 57 |
|  | **45** | 27.000 | WITHOUT CARDIAC EVENT | . | . | 5 | 56 |
|  | **46** | 28.000 | WITHOUT CARDIAC EVENT | . | . | 5 | 55 |
|  | **47** | 29.000 | WITHOUT CARDIAC EVENT | . | . | 5 | 54 |
|  | **48** | 29.000 | WITHOUT CARDIAC EVENT | . | . | 5 | 53 |
|  | **49** | 31.000 | WITHOUT CARDIAC EVENT | . | . | 5 | 52 |
|  | **50** | 31.000 | WITHOUT CARDIAC EVENT | . | . | 5 | 51 |
|  | **51** | 31.000 | WITHOUT CARDIAC EVENT | . | . | 5 | 50 |
|  | **52** | 32.000 | WITHOUT CARDIAC EVENT | . | . | 5 | 49 |
|  | **53** | 32.000 | WITHOUT CARDIAC EVENT | . | . | 5 | 48 |
|  | **54** | 32.000 | WITHOUT CARDIAC EVENT | . | . | 5 | 47 |
|  | **55** | 32.000 | WITHOUT CARDIAC EVENT | . | . | 5 | 46 |
|  | **56** | 32.000 | WITHOUT CARDIAC EVENT | . | . | 5 | 45 |
|  | **57** | 33.000 | WITHOUT CARDIAC EVENT | . | . | 5 | 44 |
|  | **58** | 34.000 | WITHOUT CARDIAC EVENT | . | . | 5 | 43 |
|  | **59** | 34.000 | WITHOUT CARDIAC EVENT | . | . | 5 | 42 |
|  | **60** | 34.000 | WITHOUT CARDIAC EVENT | . | . | 5 | 41 |
|  | **61** | 34.000 | WITHOUT CARDIAC EVENT | . | . | 5 | 40 |
|  | **62** | 36.000 | WITHOUT CARDIAC EVENT | . | . | 5 | 39 |
|  | **63** | 37.000 | WITHOUT CARDIAC EVENT | . | . | 5 | 38 |
|  | **64** | 38.000 | WITHOUT CARDIAC EVENT | . | . | 5 | 37 |
|  | **65** | 38.000 | WITHOUT CARDIAC EVENT | . | . | 5 | 36 |
|  | **66** | 38.000 | WITHOUT CARDIAC EVENT | . | . | 5 | 35 |
|  | **67** | 39.000 | WITHOUT CARDIAC EVENT | . | . | 5 | 34 |
|  | **68** | 39.000 | WITHOUT CARDIAC EVENT | . | . | 5 | 33 |
|  | **69** | 40.000 | WITHOUT CARDIAC EVENT | . | . | 5 | 32 |
|  | **70** | 41.000 | WITHOUT CARDIAC EVENT | . | . | 5 | 31 |
|  | **71** | 41.000 | WITHOUT CARDIAC EVENT | . | . | 5 | 30 |
|  | **72** | 41.000 | WITHOUT CARDIAC EVENT | . | . | 5 | 29 |
|  | **73** | 41.000 | WITHOUT CARDIAC EVENT | . | . | 5 | 28 |
|  | **74** | 41.000 | WITHOUT CARDIAC EVENT | . | . | 5 | 27 |
|  | **75** | 42.000 | WITHOUT CARDIAC EVENT | . | . | 5 | 26 |
|  | **76** | 44.000 | WITHOUT CARDIAC EVENT | . | . | 5 | 25 |
|  | **77** | 44.000 | WITHOUT CARDIAC EVENT | . | . | 5 | 24 |
|  | **78** | 45.000 | WITHOUT CARDIAC EVENT | . | . | 5 | 23 |
|  | **79** | 46.000 | WITHOUT CARDIAC EVENT | . | . | 5 | 22 |
|  | **80** | 46.000 | WITHOUT CARDIAC EVENT | . | . | 5 | 21 |
|  | **81** | 46.000 | WITHOUT CARDIAC EVENT | . | . | 5 | 20 |
|  | **82** | 47.000 | WITHOUT CARDIAC EVENT | . | . | 5 | 19 |
|  | **83** | 47.000 | WITHOUT CARDIAC EVENT | . | . | 5 | 18 |
|  | **84** | 48.000 | WITHOUT CARDIAC EVENT | . | . | 5 | 17 |
|  | **85** | 49.000 | WITHOUT CARDIAC EVENT | . | . | 5 | 16 |
|  | **86** | 49.000 | WITHOUT CARDIAC EVENT | . | . | 5 | 15 |
|  | **87** | 50.000 | WITHOUT CARDIAC EVENT | . | . | 5 | 14 |
|  | **88** | 50.000 | WITHOUT CARDIAC EVENT | . | . | 5 | 13 |
|  | **89** | 51.000 | WITHOUT CARDIAC EVENT | . | . | 5 | 12 |
|  | **90** | 52.000 | WITHOUT CARDIAC EVENT | . | . | 5 | 11 |
|  | **91** | 52.000 | WITHOUT CARDIAC EVENT | . | . | 5 | 10 |
|  | **92** | 53.000 | WITH CARDIAC EVENT | .842 | .092 | 6 | 9 |
|  | **93** | 53.000 | WITHOUT CARDIAC EVENT | . | . | 6 | 8 |
|  | **94** | 53.000 | WITHOUT CARDIAC EVENT | . | . | 6 | 7 |
|  | **95** | 53.000 | WITHOUT CARDIAC EVENT | . | . | 6 | 6 |
|  | **96** | 55.000 | WITHOUT CARDIAC EVENT | . | . | 6 | 5 |
|  | **97** | 56.000 | WITHOUT CARDIAC EVENT | . | . | 6 | 4 |
|  | **98** | 57.000 | WITHOUT CARDIAC EVENT | . | . | 6 | 3 |
|  | **99** | 62.000 | WITHOUT CARDIAC EVENT | . | . | 6 | 2 |
|  | **100** | 64.000 | WITHOUT CARDIAC EVENT | . | . | 6 | 1 |
|  | **101** | 65.000 | WITHOUT CARDIAC EVENT | . | . | 6 | 0 |

| Means and Medians for Survival Time | | | | | | | | |
| --- | --- | --- | --- | --- | --- | --- | --- | --- |
| **EE normal/ischemic** | **Mean(a)** | | | | **Median** | | | |
|  | **Estimate** | **Std. Error** | **95% Confidence Interval** | | **Estimate** | **Std. Error** | **95% Confidence Interval** | |
|  |  |  | **Lower Bound** | **Upper Bound** |  |  | **Lower Bound** | **Upper Bound** |
| **G1 – Ischemic** | 53.115 | 2.442 | 48.329 | 57.902 | . | . | . | . |
| **G2 – Normal** | 60.971 | 1.625 | 57.785 | 64.157 | . | . | . | . |
| **Overall** | 57.299 | 1.447 | 54.463 | 60.135 | . | . | . | . |
| a Estimation is limited to the largest survival time if it is censored. | | | | | | | | |

| Overall Comparisons | | | |
| --- | --- | --- | --- |
|  | **Chi-Square** | **df** | **Sig.** |
| **Log Rank (Mantel-Cox)** | 8.345 | 1 | .004 |
| **Breslow (Generalized Wilcoxon)** | 8.882 | 1 | .003 |
| Test of equality of survival distributions for the different levels of EE result. | | | |

Kaplan-Meier

| Notes | | |
| --- | --- | --- |
| **Output Created** | | 28-MAR-2008 07:44:26 |
| **Comments** | |  |
| **Input** | **Data** | C:\Documents and Settings\ENALDO\My Documents\CALCULOS ESTATISTICOS\TRAB JOSELINA E TAUANE\NATHALIE 13032008\DIABETES140308.sav |
|  | **Filter** | <none> |
|  | **Weight** | <none> |
|  | **Split File** | <none> |
|  | **N of Rows in Working Data File** | 193 |
| **Missing Value Handling** | **Definition of Missing** | User-defined missing values are treated as missing. |
|  | **Cases Used** | Statistics are based on all cases with valid data for all variables in the analysis. |
| **Syntax** | | KM TEVENTO BY sedentary /STATUS=CENSOR(1) /PRINT TABLE MEAN /PLOT SURVIVAL /TEST LOGRANK BRESLOW /COMPARE OVERALL POOLED /SAVE SURVIVAL CUMEVENT . |
| **Resources** | **Elapsed Time** | 0:00:00.75 |
| **Variables Created or Modified** | **SUR_2** | Survival function |
|  | **CUM_2** | Cumulative number of events |

| Case Processing Summary | | | | |
| --- | --- | --- | --- | --- |
| **SEDENTARY LIFESTYLE** | **Total N** | **N of Events** | **Censored** | |
|  |  |  | **N** | **Percent** |
| **Sedentary** | 90 | 16 | 74 | 82.2% |
| **Non-sedentary** | 103 | 8 | 95 | 92.2% |
| **Overall** | 193 | 24 | 169 | 87.6% |

| Survival Table | | | | | | | |
| --- | --- | --- | --- | --- | --- | --- | --- |
| **SEDENTARY LIFESTYLE** |  | **Time** | **Status** | **Cumulative Proportion Surviving at the Time** | | **N of Cumulative Events** | **N of Remaining Cases** |
|  |  |  |  | **Estimate** | **Std. Error** |  |  |
| **SEDENTARY** | **1** | 7.000 | WITH CARDIAC EVENT | .989 | .011 | 1 | 89 |
|  | **2** | 7.000 | WITHOUT CARDIAC EVENT | . | . | 1 | 88 |
|  | **3** | 7.000 | WITHOUT CARDIAC EVENT | . | . | 1 | 87 |
|  | **4** | 8.000 | WITHOUT CARDIAC EVENT | . | . | 1 | 86 |
|  | **5** | 9.000 | WITH CARDIAC EVENT | .977 | .016 | 2 | 85 |
|  | **6** | 10.000 | WITH CARDIAC EVENT | .966 | .019 | 3 | 84 |
|  | **7** | 10.000 | WITHOUT CARDIAC EVENT | . | . | 3 | 83 |
|  | **8** | 10.000 | WITHOUT CARDIAC EVENT | . | . | 3 | 82 |
|  | **9** | 11.000 | WITHOUT CARDIAC EVENT | . | . | 3 | 81 |
|  | **10** | 11.000 | WITHOUT CARDIAC EVENT | . | . | 3 | 80 |
|  | **11** | 12.000 | WITH CARDIAC EVENT | .954 | .023 | 4 | 79 |
|  | **12** | 12.000 | WITHOUT CARDIAC EVENT | . | . | 4 | 78 |
|  | **13** | 12.000 | WITHOUT CARDIAC EVENT | . | . | 4 | 77 |
|  | **14** | 12.000 | WITHOUT CARDIAC EVENT | . | . | 4 | 76 |
|  | **15** | 13.000 | WITH CARDIAC EVENT | .941 | .026 | 5 | 75 |
|  | **16** | 13.000 | WITHOUT CARDIAC EVENT | . | . | 5 | 74 |
|  | **17** | 14.000 | WITH CARDIAC EVENT | .929 | .028 | 6 | 73 |
|  | **18** | 14.000 | WITHOUT CARDIAC EVENT | . | . | 6 | 72 |
|  | **19** | 14.000 | WITHOUT CARDIAC EVENT | . | . | 6 | 71 |
|  | **20** | 15.000 | WITH CARDIAC EVENT | . | . | 7 | 70 |
|  | **21** | 15.000 | WITH CARDIAC EVENT | .902 | .033 | 8 | 69 |
|  | **22** | 15.000 | WITHOUT CARDIAC EVENT | . | . | 8 | 68 |
|  | **23** | 16.000 | WITHOUT CARDIAC EVENT | . | . | 8 | 67 |
|  | **24** | 17.000 | WITHOUT CARDIAC EVENT | . | . | 8 | 66 |
|  | **25** | 17.000 | WITHOUT CARDIAC EVENT | . | . | 8 | 65 |
|  | **26** | 19.000 | WITHOUT CARDIAC EVENT | . | . | 8 | 64 |
|  | **27** | 19.000 | WITHOUT CARDIAC EVENT | . | . | 8 | 63 |
|  | **28** | 20.000 | WITHOUT CARDIAC EVENT | . | . | 8 | 62 |
|  | **29** | 20.000 | WITHOUT CARDIAC EVENT | . | . | 8 | 61 |
|  | **30** | 20.000 | WITHOUT CARDIAC EVENT | . | . | 8 | 60 |
|  | **31** | 21.000 | WITH CARDIAC EVENT | .887 | .036 | 9 | 59 |
|  | **32** | 21.000 | WITHOUT CARDIAC EVENT | . | . | 9 | 58 |
|  | **33** | 22.000 | WITHOUT CARDIAC EVENT | . | . | 9 | 57 |
|  | **34** | 22.000 | WITHOUT CARDIAC EVENT | . | . | 9 | 56 |
|  | **35** | 23.000 | WITHOUT CARDIAC EVENT | . | . | 9 | 55 |
|  | **36** | 23.000 | WITHOUT CARDIAC EVENT | . | . | 9 | 54 |
|  | **37** | 24.000 | WITHOUT CARDIAC EVENT | . | . | 9 | 53 |
|  | **38** | 24.000 | WITHOUT CARDIAC EVENT | . | . | 9 | 52 |
|  | **39** | 24.000 | WITHOUT CARDIAC EVENT | . | . | 9 | 51 |
|  | **40** | 24.000 | WITHOUT CARDIAC EVENT | . | . | 9 | 50 |
|  | **41** | 25.000 | WITH CARDIAC EVENT | .870 | .039 | 10 | 49 |
|  | **42** | 25.000 | WITHOUT CARDIAC EVENT | . | . | 10 | 48 |
|  | **43** | 25.000 | WITHOUT CARDIAC EVENT | . | . | 10 | 47 |
|  | **44** | 25.000 | WITHOUT CARDIAC EVENT | . | . | 10 | 46 |
|  | **45** | 25.000 | WITHOUT CARDIAC EVENT | . | . | 10 | 45 |
|  | **46** | 26.000 | WITHOUT CARDIAC EVENT | . | . | 10 | 44 |
|  | **47** | 27.000 | WITH CARDIAC EVENT | .850 | .043 | 11 | 43 |
|  | **48** | 27.000 | WITHOUT CARDIAC EVENT | . | . | 11 | 42 |
|  | **49** | 28.000 | WITH CARDIAC EVENT | .830 | .046 | 12 | 41 |
|  | **50** | 28.000 | WITHOUT CARDIAC EVENT | . | . | 12 | 40 |
|  | **51** | 29.000 | WITH CARDIAC EVENT | .809 | .050 | 13 | 39 |
|  | **52** | 29.000 | WITHOUT CARDIAC EVENT | . | . | 13 | 38 |
|  | **53** | 29.000 | WITHOUT CARDIAC EVENT | . | . | 13 | 37 |
|  | **54** | 31.000 | WITHOUT CARDIAC EVENT | . | . | 13 | 36 |
|  | **55** | 31.000 | WITHOUT CARDIAC EVENT | . | . | 13 | 35 |
|  | **56** | 31.000 | WITHOUT CARDIAC EVENT | . | . | 13 | 34 |
|  | **57** | 32.000 | WITHOUT CARDIAC EVENT | . | . | 13 | 33 |
|  | **58** | 32.000 | WITHOUT CARDIAC EVENT | . | . | 13 | 32 |
|  | **59** | 32.000 | WITHOUT CARDIAC EVENT | . | . | 13 | 31 |
|  | **60** | 34.000 | WITHOUT CARDIAC EVENT | . | . | 13 | 30 |
|  | **61** | 35.000 | WITH CARDIAC EVENT | .782 | .055 | 14 | 29 |
|  | **62** | 37.000 | WITHOUT CARDIAC EVENT | . | . | 14 | 28 |
|  | **63** | 37.000 | WITHOUT CARDIAC EVENT | . | . | 14 | 27 |
|  | **64** | 38.000 | WITH CARDIAC EVENT | .753 | .060 | 15 | 26 |
|  | **65** | 38.000 | WITHOUT CARDIAC EVENT | . | . | 15 | 25 |
|  | **66** | 38.000 | WITHOUT CARDIAC EVENT | . | . | 15 | 24 |
|  | **67** | 39.000 | WITHOUT CARDIAC EVENT | . | . | 15 | 23 |
|  | **68** | 40.000 | WITHOUT CARDIAC EVENT | . | . | 15 | 22 |
|  | **69** | 40.000 | WITHOUT CARDIAC EVENT | . | . | 15 | 21 |
|  | **70** | 42.000 | WITHOUT CARDIAC EVENT | . | . | 15 | 20 |
|  | **71** | 43.000 | WITHOUT CARDIAC EVENT | . | . | 15 | 19 |
|  | **72** | 44.000 | WITHOUT CARDIAC EVENT | . | . | 15 | 18 |
|  | **73** | 45.000 | WITHOUT CARDIAC EVENT | . | . | 15 | 17 |
|  | **74** | 46.000 | WITHOUT CARDIAC EVENT | . | . | 15 | 16 |
|  | **75** | 47.000 | WITHOUT CARDIAC EVENT | . | . | 15 | 15 |
|  | **76** | 47.000 | WITHOUT CARDIAC EVENT | . | . | 15 | 14 |
|  | **77** | 48.000 | WITHOUT CARDIAC EVENT | . | . | 15 | 13 |
|  | **78** | 48.000 | WITHOUT CARDIAC EVENT | . | . | 15 | 12 |
|  | **79** | 49.000 | WITHOUT CARDIAC EVENT | . | . | 15 | 11 |
|  | **80** | 49.000 | WITHOUT CARDIAC EVENT | . | . | 15 | 10 |
|  | **81** | 52.000 | WITHOUT CARDIAC EVENT | . | . | 15 | 9 |
|  | **82** | 52.000 | WITHOUT CARDIAC EVENT | . | . | 15 | 8 |
|  | **83** | 53.000 | WITHOUT CARDIAC EVENT | .659 | .102 | 16 | 7 |
|  | **84** | 53.000 | WITHOUT CARDIAC EVENT | . | . | 16 | 6 |
|  | **85** | 56.000 | WITHOUT CARDIAC EVENT | . | . | 16 | 5 |
|  | **86** | 56.000 | WITHOUT CARDIAC EVENT | . | . | 16 | 4 |
|  | **87** | 57.000 | WITHOUT CARDIAC EVENT | . | . | 16 | 3 |
|  | **88** | 57.000 | WITHOUT CARDIAC EVENT | . | . | 16 | 2 |
|  | **89** | 57.000 | WITHOUT CARDIAC EVENT | . | . | 16 | 1 |
|  | **90** | 57.000 | WITHOUT CARDIAC EVENT | . | . | 16 | 0 |
| **NON-SEDENTARY** | **1** | 7.000 | WITH CARDIAC EVENT | .990 | .010 | 1 | 102 |
|  | **2** | 7.000 | WITHOUT CARDIAC EVENT | . | . | 1 | 101 |
|  | **3** | 7.000 | WITHOUT CARDIAC EVENT | . | . | 1 | 100 |
|  | **4** | 7.000 | WITHOUT CARDIAC EVENT | . | . | 1 | 99 |
|  | **5** | 7.000 | WITHOUT CARDIAC EVENT | . | . | 1 | 98 |
|  | **6** | 7.000 | WITHOUT CARDIAC EVENT | . | . | 1 | 97 |
|  | **7** | 9.000 | WITHOUT CARDIAC EVENT | . | . | 1 | 96 |
|  | **8** | 9.000 | WITHOUT CARDIAC EVENT | . | . | 1 | 95 |
|  | **9** | 9.000 | WITHOUT CARDIAC EVENT | . | . | 1 | 94 |
|  | **10** | 9.000 | WITHOUT CARDIAC EVENT | . | . | 1 | 93 |
|  | **11** | 10.000 | WITH CARDIAC EVENT | .980 | .014 | 2 | 92 |
|  | **12** | 10.000 | WITHOUT CARDIAC EVENT | . | . | 2 | 91 |
|  | **13** | 10.000 | WITHOUT CARDIAC EVENT | . | . | 2 | 90 |
|  | **14** | 11.000 | WITH CARDIAC EVENT | .969 | .018 | 3 | 89 |
|  | **15** | 11.000 | WITHOUT CARDIAC EVENT | . | . | 3 | 88 |
|  | **16** | 11.000 | WITHOUT CARDIAC EVENT | . | . | 3 | 87 |
|  | **17** | 12.000 | WITH CARDIAC EVENT | .958 | .021 | 4 | 86 |
|  | **18** | 12.000 | WITHOUT CARDIAC EVENT | . | . | 4 | 85 |
|  | **19** | 14.000 | WITH CARDIAC EVENT | .946 | .023 | 5 | 84 |
|  | **20** | 14.000 | WITHOUT CARDIAC EVENT | . | . | 5 | 83 |
|  | **21** | 15.000 | WITHOUT CARDIAC EVENT | . | . | 5 | 82 |
|  | **22** | 15.000 | WITHOUT CARDIAC EVENT | . | . | 5 | 81 |
|  | **23** | 15.000 | WITHOUT CARDIAC EVENT | . | . | 5 | 80 |
|  | **24** | 16.000 | WITHOUT CARDIAC EVENT | . | . | 5 | 79 |
|  | **25** | 16.000 | WITHOUT CARDIAC EVENT | . | . | 5 | 78 |
|  | **26** | 16.000 | WITHOUT CARDIAC EVENT | . | . | 5 | 77 |
|  | **27** | 17.000 | WITHOUT CARDIAC EVENT | . | . | 5 | 76 |
|  | **28** | 18.000 | WITHOUT CARDIAC EVENT | . | . | 5 | 75 |
|  | **29** | 18.000 | WITHOUT CARDIAC EVENT | . | . | 5 | 74 |
|  | **30** | 18.000 | WITHOUT CARDIAC EVENT | . | . | 5 | 73 |
|  | **31** | 19.000 | WITHOUT CARDIAC EVENT | . | . | 5 | 72 |
|  | **32** | 20.000 | WITH CARDIAC EVENT | .933 | .026 | 6 | 71 |
|  | **33** | 20.000 | WITHOUT CARDIAC EVENT | . | . | 6 | 70 |
|  | **34** | 20.000 | WITHOUT CARDIAC EVENT | . | . | 6 | 69 |
|  | **35** | 21.000 | WITHOUT CARDIAC EVENT | . | . | 6 | 68 |
|  | **36** | 21.000 | WITHOUT CARDIAC EVENT | . | . | 6 | 67 |
|  | **37** | 21.000 | WITHOUT CARDIAC EVENT | . | . | 6 | 66 |
|  | **38** | 22.000 | WITHOUT CARDIAC EVENT | . | . | 6 | 65 |
|  | **39** | 23.000 | WITHOUT CARDIAC EVENT | . | . | 6 | 64 |
|  | **40** | 24.000 | WITHOUT CARDIAC EVENT | . | . | 6 | 63 |
|  | **41** | 24.000 | WITHOUT CARDIAC EVENT | . | . | 6 | 62 |
|  | **42** | 24.000 | WITHOUT CARDIAC EVENT | . | . | 6 | 61 |
|  | **43** | 25.000 | WITHOUT CARDIAC EVENT | . | . | 6 | 60 |
|  | **44** | 25.000 | WITHOUT CARDIAC EVENT | . | . | 6 | 59 |
|  | **45** | 27.000 | WITHOUT CARDIAC EVENT | . | . | 6 | 58 |
|  | **46** | 27.000 | WITHOUT CARDIAC EVENT | . | . | 6 | 57 |
|  | **47** | 29.000 | WITHOUT CARDIAC EVENT | . | . | 6 | 56 |
|  | **48** | 29.000 | WITHOUT CARDIAC EVENT | . | . | 6 | 55 |
|  | **49** | 31.000 | WITHOUT CARDIAC EVENT | . | . | 6 | 54 |
|  | **50** | 32.000 | WITHOUT CARDIAC EVENT | . | . | 6 | 53 |
|  | **51** | 32.000 | WITHOUT CARDIAC EVENT | . | . | 6 | 52 |
|  | **52** | 32.000 | WITHOUT CARDIAC EVENT | . | . | 6 | 51 |
|  | **53** | 32.000 | WITHOUT CARDIAC EVENT | . | . | 6 | 50 |
|  | **54** | 33.000 | WITHOUT CARDIAC EVENT | . | . | 6 | 49 |
|  | **55** | 34.000 | WITHOUT CARDIAC EVENT | . | . | 6 | 48 |
|  | **56** | 34.000 | WITHOUT CARDIAC EVENT | . | . | 6 | 47 |
|  | **57** | 34.000 | WITHOUT CARDIAC EVENT | . | . | 6 | 46 |
|  | **58** | 35.000 | WITHOUT CARDIAC EVENT | . | . | 6 | 45 |
|  | **59** | 36.000 | WITHOUT CARDIAC EVENT | . | . | 6 | 44 |
|  | **60** | 38.000 | WITHOUT CARDIAC EVENT | . | . | 6 | 43 |
|  | **61** | 39.000 | WITH CARDIAC EVENT | .912 | .034 | 7 | 42 |
|  | **62** | 39.000 | WITHOUT CARDIAC EVENT | . | . | 7 | 41 |
|  | **63** | 39.000 | WITHOUT CARDIAC EVENT | . | . | 7 | 40 |
|  | **64** | 39.000 | WITHOUT CARDIAC EVENT | . | . | 7 | 39 |
|  | **65** | 40.000 | WITHOUT CARDIAC EVENT | . | . | 7 | 38 |
|  | **66** | 41.000 | WITHOUT CARDIAC EVENT | . | . | 7 | 37 |
|  | **67** | 41.000 | WITHOUT CARDIAC EVENT | . | . | 7 | 36 |
|  | **68** | 41.000 | WITHOUT CARDIAC EVENT | . | . | 7 | 35 |
|  | **69** | 41.000 | WITHOUT CARDIAC EVENT | . | . | 7 | 34 |
|  | **70** | 41.000 | WITHOUT CARDIAC EVENT | . | . | 7 | 33 |
|  | **71** | 41.000 | WITHOUT CARDIAC EVENT | . | . | 7 | 32 |
|  | **72** | 42.000 | WITHOUT CARDIAC EVENT | . | . | 7 | 31 |
|  | **73** | 42.000 | WITHOUT CARDIAC EVENT | . | . | 7 | 30 |
|  | **74** | 42.000 | WITHOUT CARDIAC EVENT | . | . | 7 | 29 |
|  | **75** | 43.000 | WITH CARDIAC EVENT | .880 | .045 | 8 | 28 |
|  | **76** | 44.000 | WITHOUT CARDIAC EVENT | . | . | 8 | 27 |
|  | **77** | 46.000 | WITHOUT CARDIAC EVENT | . | . | 8 | 26 |
|  | **78** | 46.000 | WITHOUT CARDIAC EVENT | . | . | 8 | 25 |
|  | **79** | 46.000 | WITHOUT CARDIAC EVENT | . | . | 8 | 24 |
|  | **80** | 47.000 | WITHOUT CARDIAC EVENT | . | . | 8 | 23 |
|  | **81** | 49.000 | WITHOUT CARDIAC EVENT | . | . | 8 | 22 |
|  | **82** | 50.000 | WITHOUT CARDIAC EVENT | . | . | 8 | 21 |
|  | **83** | 50.000 | WITHOUT CARDIAC EVENT | . | . | 8 | 20 |
|  | **84** | 50.000 | WITHOUT CARDIAC EVENT | . | . | 8 | 19 |
|  | **85** | 50.000 | WITHOUT CARDIAC EVENT | . | . | 8 | 18 |
|  | **86** | 51.000 | WITHOUT CARDIAC EVENT | . | . | 8 | 17 |
|  | **87** | 52.000 | WITHOUT CARDIAC EVENT | . | . | 8 | 16 |
|  | **88** | 53.000 | WITHOUT CARDIAC EVENT | . | . | 8 | 15 |
|  | **89** | 53.000 | WITHOUT CARDIAC EVENT | . | . | 8 | 14 |
|  | **90** | 53.000 | WITHOUT CARDIAC EVENT | . | . | 8 | 13 |
|  | **91** | 55.000 | WITHOUT CARDIAC EVENT | . | . | 8 | 12 |
|  | **92** | 55.000 | WITHOUT CARDIAC EVENT | . | . | 8 | 11 |
|  | **93** | 56.000 | WITHOUT CARDIAC EVENT | . | . | 8 | 10 |
|  | **94** | 56.000 | WITHOUT CARDIAC EVENT | . | . | 8 | 9 |
|  | **95** | 58.000 | WITHOUT CARDIAC EVENT | . | . | 8 | 8 |
|  | **96** | 58.000 | WITHOUT CARDIAC EVENT | . | . | 8 | 7 |
|  | **97** | 60.000 | WITHOUT CARDIAC EVENT | . | . | 8 | 6 |
|  | **98** | 61.000 | WITHOUT CARDIAC EVENT | . | . | 8 | 5 |
|  | **99** | 62.000 | WITHOUT CARDIAC EVENT | . | . | 8 | 4 |
|  | **100** | 62.000 | WITHOUT CARDIAC EVENT | . | . | 8 | 3 |
|  | **101** | 64.000 | WITHOUT CARDIAC EVENT | . | . | 8 | 2 |
|  | **102** | 65.000 | WITHOUT CARDIAC EVENT | . | . | 8 | 1 |
|  | **103** | 65.000 | WITHOUT CARDIAC EVENT | . | . | 8 | 0 |

| Means and Medians for Survival Time | | | | | | | | |
| --- | --- | --- | --- | --- | --- | --- | --- | --- |
| **SEDENTARY LIFESTYLE** | **Mean(a)** | | | | **Median** | | | |
|  | **Estimate** | **Std. Error** | **95% Confidence Interval** | | **Estimate** | **Std. Error** | **95% Confidence Interval** | |
|  |  |  | **Lower Bound** | **Upper Bound** |  |  | **Lower Bound** | **Upper Bound** |
| **Sedentary** | 48.221 | 1.936 | 44.426 | 52.017 | . | . | . | . |
| **Non-sedentary** | 60.251 | 1.601 | 57.114 | 63.389 | . | . | . | . |
| **Overall** | 57.299 | 1.447 | 54.463 | 60.135 | . | . | . | . |
| a Estimation is limited to the largest survival time if it is censored. | | | | | | | | |

| Overall Comparisons | | | |
| --- | --- | --- | --- |
|  | **Chi-Square** | **df** | **Sig.** |
| **Log Rank (Mantel-Cox)** | 4.899 | 1 | **.03** |
| **Breslow (Generalized Wilcoxon)** | 3.131 | 1 | .077 |
| Test of equality of survival distributions for the different levels of SEDENTARISMO. | | | |

**Descriptives**

| Gender | | | | | |
| --- | --- | --- | --- | --- | --- |
|  |  | **Frequency** | **Percent** | **Valid Percent** | **Cumulative Percent** |
| **Valid** | **Female** | 96 | 49.7 | 49.7 | 49.7 |
|  | **Male** | 97 | 50.3 | 50.3 | 100.0 |
|  | **Total** | 193 | 100.0 | 100.0 |  |

| Descriptive Statistics | | | | | |
| --- | --- | --- | --- | --- | --- |
|  | **N** | **Minimum** | **Maximum** | **Mean** | **Std. Deviation** |
| **Patient's age** | 192 | **38.0** | **91.0** | **59.8** | **9.3** |
| **Valid N (listwise)** | 192 |  |  |  |  |

| Insulin takers | | | | | |
| --- | --- | --- | --- | --- | --- |
|  |  | **Frequency** | **Percent** | **Valid Percent** | **Cumulative Percent** |
| **Valid** | **False** | 141 | 73.1 | 73.1 | 73.1 |
|  | **true** | 52 | 26.9 | 26.9 | 100.0 |
|  | **Total** | 193 | 100.0 | 100.0 |  |

| Percutaneous and surgical revascularization before EE | | | | | |
| --- | --- | --- | --- | --- | --- |
|  |  | **Frequency** | **Percent** | **Valid Percent** | **Cumulative Percent** |
| **Valid** | **False** | 147 | 76.2 | 76.2 | 76.2 |
|  | **True** | 43 | 22.3 | 22.3 | 98.4 |
|  | **True surgical+percutaneous** | 3 | 1.6 | 1.6 | 100.0 |
|  | **Total** | 193 | 100.0 | 100.0 |  |

| OLD AND RECENT MYOCARDIAL INFARCTION BEFORE EE | | | | | |
| --- | --- | --- | --- | --- | --- |
|  |  | **Frequency** | **Percent** | **Valid Percent** | **Cumulative Percent** |
| **Valid** | **False** | 165 | 85.5 | 85.5 | 85.5 |
|  | **True** | 28 | 14.5 | 14.5 | 100.0 |
|  | **Total** | 193 | 100.0 | 100.0 |  |

| HYPERTENSION | | | | | |
| --- | --- | --- | --- | --- | --- |
|  |  | **Frequency** | **Percent** | **Valid Percent** | **Cumulative Percent** |
| **Valid** | **False** | 22 | 11.4 | 11.4 | 11.4 |
|  | **True** | 171 | 88.6 | 88.6 | 100.0 |
|  | **Total** | 193 | 100.0 | 100.0 |  |

| DYSLIPIDEMIA | | | | | |
| --- | --- | --- | --- | --- | --- |
|  |  | **Frequency** | **Percent** | **Valid Percent** | **Cumulative Percent** |
| **Valid** | **False** | 75 | 38.9 | 38.9 | 38.9 |
|  | **True** | 118 | 61.1 | 61.1 | 100.0 |
|  | **Total** | 193 | 100.0 | 100.0 |  |

| SEDENTARY LIFESTYLE | | | | | |
| --- | --- | --- | --- | --- | --- |
|  |  | **Frequency** | **Percent** | **Valid Percent** | **Cumulative Percent** |
| **Valid** | **Sedentary** | 90 | 46.6 | 46.6 | 46.6 |
|  | **Non-sedentary** | 103 | 53.4 | 53.4 | 100.0 |
|  | **Total** | 193 | 100.0 | 100.0 |  |

T-Test

| Notes | | |
| --- | --- | --- |
| **Output Created** | | 12-MAR-2008 11:13:05 |
| **Comments** | |  |
| **Input** | **Data** | C:\Documents and Settings\Joselina Oliveira\Desktop\ECOESTRESSE\THAIANA DIABETES\THAIANA 140807\BCO DIABETES PRECORDIALGIA censurada 13082007.sav |
|  | **Filter** | <none> |
|  | **Weight** | <none> |
|  | **Split File** | <none> |
|  | **N of Rows in Working Data File** | 193 |
| **Missing Value Handling** | **Definition of Missing** | User defined missing values are treated as missing. |
|  | **Cases Used** | Statistics for each analysis are based on the cases with no missing or out-of-range data for any variable in the analysis. |
| **Syntax** | | T-TEST GROUPS = eeefni(1 2) /MISSING = ANALYSIS /VARIABLES = idade peso altura /CRITERIA = CI(.95) . |
| **Resources** | **Elapsed Time** | 0:00:00,05 |

| Group Statistics | | | | | |
| --- | --- | --- | --- | --- | --- |
|  | **EE result** | **N** | **Mean** | **Std. Deviation** | **Std. Error Mean** |
| **Patient's age** | **Normal** | 101 | 59,3168 | 9,09278 | ,90477 |
|  | **Ischemic** | 91 | 60,3956 | 9,50424 | ,99632 |

| Independent Samples Test | | | | | | | | | | |
| --- | --- | --- | --- | --- | --- | --- | --- | --- | --- | --- |
|  |  | **Levene's Test for Equality of Variances** | | **t-test for Equality of Means** | | | | | | |
|  |  | **F** | **Sig.** | **t** | **df** | **Sig. (2-tailed)** | **Mean Difference** | **Std. Error Difference** | **95% Confidence Interval of the Difference** | |
|  |  |  |  |  |  |  |  |  | **Lower** | **Upper** |
| **Patient's age** | **Equal variances assumed** | ,104 | ,747 | -,803 | 190 | ,423 | -1,07877 | 1,34271 | -3,72731 | 1,56976 |
|  | **Equal variances not assumed** |  |  | -,802 | 185,877 | ,424 | -1,07877 | 1,34582 | -3,73383 | 1,57628 |

**Crosstabs**

**Gender * EE result**

| Crosstab | | | | | |
| --- | --- | --- | --- | --- | --- |
|  |  |  | **EE result** | | **Total** |
|  |  |  | **G1 – Ischemic** | **G2 - Normal** |  |
| **Gender** | **Feminine** | **Count** | 48 | 48 | 96 |
|  |  | **% within EE result** | 52.2% | 47.5% | 49.7% |
|  |  | **% of Total** | 24.9% | 24.9% | 49.7% |
|  | **Masculine** | **Count** | 44 | 53 | 97 |
|  |  | **% within EE result** | **47.8%** | **52.5%** | 50.3% |
|  |  | **% of Total** | 22.8% | 27.5% | 50.3% |
| **Total** | | **Count** | 92 | 101 | 193 |
|  |  | **% within EE result** | 100.0% | 100.0% | 100.0% |
|  |  | **% of Total** | 47.7% | 52.3% | 100.0% |

| Chi-Square Tests | | | | | |
| --- | --- | --- | --- | --- | --- |
|  | **Value** | **df** | **Asymp. Sig. (2-sided)** | **Exact Sig. (2-sided)** | **Exact Sig. (1-sided)** |
| **Pearson Chi-Square** | .416(b) | 1 | **.52** |  |  |
| **Continuity Correction(a)** | .251 | 1 | .616 |  |  |
| **Likelihood Ratio** | .416 | 1 | .519 |  |  |
| **Fisher's Exact Test** |  |  |  | .566 | .308 |
| **Linear-by-Linear Association** | .414 | 1 | .520 |  |  |
| **N of Valid Cases** | 193 |  |  |  |  |

**Hypertension * EE result**

| Crosstab | | | | | |
| --- | --- | --- | --- | --- | --- |
|  |  |  | **EE result** | | **Total** |
|  |  |  | **G1 - Ischemic** | **G2 - Normal** |  |
| **Hypertension** | **False** | **Count** | 11 | 11 | 22 |
|  |  | **% within EE result** | 12.0% | 10.9% | 11.4% |
|  |  | **% of Total** | 5.7% | 5.7% | 11.4% |
|  | **True** | **Count** | 81 | 90 | 171 |
|  |  | **% within EE result** | **88.0%** | **89.1%** | 88.6% |
|  |  | **% of Total** | 42.0% | 46.6% | 88.6% |
| **Total** | | **Count** | 92 | 101 | 193 |
|  |  | **% within EE result** | 100.0% | 100.0% | 100.0% |
|  |  | **% of Total** | 47.7% | 52.3% | 100.0% |

| Chi-Square Tests | | | | | |
| --- | --- | --- | --- | --- | --- |
|  | **Value** | **df** | **Asymp. Sig. (2-sided)** | **Exact Sig. (2-sided)** | **Exact Sig. (1-sided)** |
| **Pearson Chi-Square** | .054(b) | 1 | **.82** |  |  |
| **Continuity Correction(a)** | .000 | 1 | .995 |  |  |
| **Likelihood Ratio** | .054 | 1 | .816 |  |  |
| **Fisher's Exact Test** |  |  |  | .825 | .497 |
| **Linear-by-Linear Association** | .054 | 1 | .817 |  |  |
| **N of Valid Cases** | 193 |  |  |  |  |

**Dyslipidemia * EE result**

| Crosstab | | | | | |
| --- | --- | --- | --- | --- | --- |
|  |  |  | **EE result** | | **Total** |
|  |  |  | **G1 - Ischemic** | **G2 - Normal** |  |
| **Dyslipidemia** | **False** | **Count** | 35 | 40 | 75 |
|  |  | **% within EE result** | 38.0% | 39.6% | 38.9% |
|  |  | **% of Total** | 18.1% | 20.7% | 38.9% |
|  | **True** | **Count** | 57 | 61 | 118 |
|  |  | **% within EE result** | **62.0%** | **60.4%** | 61.1% |
|  |  | **% of Total** | 29.5% | 31.6% | 61.1% |
| **Total** | | **Count** | 92 | 101 | 193 |
|  |  | **% within EE result** | 100.0% | 100.0% | 100.0% |
|  |  | **% of Total** | 47.7% | 52.3% | 100.0% |

| Chi-Square Tests | | | | | |
| --- | --- | --- | --- | --- | --- |
|  | **Value** | **df** | **Asymp. Sig. (2-sided)** | **Exact Sig. (2-sided)** | **Exact Sig. (1-sided)** |
| **Pearson Chi-Square** | .049(b) | 1 | .824 |  |  |
| **Continuity Correction(a)** | .006 | 1 | .941 |  |  |
| **Likelihood Ratio** | .049 | 1 | .824 |  |  |
| **Fisher's Exact Test** |  |  |  | .883 | .471 |
| **Linear-by-Linear Association** | .049 | 1 | .825 |  |  |
| **N of Valid Cases** | 193 |  |  |  |  |

**Smoking * EE result**

| Crosstab | | | | | |
| --- | --- | --- | --- | --- | --- |
|  |  |  | **EE result** | | **Total** |
|  |  |  | **G1 - Ischemic** | **G2 - Normal** |  |
| **Smoking** | **False** | **Count** | 70 | 82 | 152 |
|  |  | **% within EE result** | 76.1% | 81.2% | 78.8% |
|  |  | **% of Total** | 36.3% | 42.5% | 78.8% |
|  | **True** | **Count** | 22 | 19 | 41 |
|  |  | **% within EE result** | 23.9% | 18.8% | 21.2% |
|  |  | **% of Total** | 11.4% | 9.8% | 21.2% |
| **Total** | | **Count** | 92 | 101 | 193 |
|  |  | **% within EE result** | 100.0% | 100.0% | 100.0% |
|  |  | **% of Total** | 47.7% | 52.3% | 100.0% |

| Chi-Square Tests | | | | | |
| --- | --- | --- | --- | --- | --- |
|  | **Value** | **df** | **Asymp. Sig. (2-sided)** | **Exact Sig. (2-sided)** | **Exact Sig. (1-sided)** |
| **Pearson Chi-Square** | .749(b) | 1 | **.39** |  |  |
| **Continuity Correction(a)** | .475 | 1 | .491 |  |  |
| **Likelihood Ratio** | .748 | 1 | .387 |  |  |
| **Fisher's Exact Test** |  |  |  | .481 | .245 |
| **Linear-by-Linear Association** | .745 | 1 | .388 |  |  |
| **N of Valid Cases** | 193 |  |  |  |  |

**Sedentary lifestyle * EE result**

| Crosstab | | | | | |
| --- | --- | --- | --- | --- | --- |
|  |  |  | **EE result** | | **Total** |
|  |  |  | **G1 - Ischemic** | **G2 - Normal** |  |
| **Sedentary lifestyle** | **Sedentary** | **Count** | 45 | 45 | 90 |
|  |  | **% within EE result** | **48.9%** | **44.6%** | 46.6% |
|  |  | **% of Total** | 23.3% | 23.3% | 46.6% |
|  | **Non-sedentary** | **Count** | 47 | 56 | 103 |
|  |  | **% within EE result** | 51.1% | 55.4% | 53.4% |
|  |  | **% of Total** | 24.4% | 29.0% | 53.4% |
| **Total** | | **Count** | 92 | 101 | 193 |
|  |  | **% within EE result** | 100.0% | 100.0% | 100.0% |
|  |  | **% of Total** | 47.7% | 52.3% | 100.0% |

| Chi-Square Tests | | | | | |
| --- | --- | --- | --- | --- | --- |
|  | **Value** | **df** | **Asymp. Sig. (2-sided)** | **Exact Sig. (2-sided)** | **Exact Sig. (1-sided)** |
| **Pearson Chi-Square** | .368(b) | 1 | **.54** |  |  |
| **Continuity Correction(a)** | .213 | 1 | .644 |  |  |
| **Likelihood Ratio** | .368 | 1 | .544 |  |  |
| **Fisher's Exact Test** |  |  |  | .566 | .322 |
| **Linear-by-Linear Association** | .366 | 1 | .545 |  |  |
| **N of Valid Cases** | 193 |  |  |  |  |

**BMI (body mass index)**

| Group Statistics | | | | | |
| --- | --- | --- | --- | --- | --- |
|  | **EE result** | **N** | **Mean** | **Std. Deviation** | **Std. Error Mean** |
| **BMI** | **G1 – Ischemic** | 90 | **27.9** | **4.2** | .44050 |
|  | **G2 – Normal** | 101 | **28.9** | **3.8** | .37894 |

| Independent Samples Test | | | | | | | | | | |
| --- | --- | --- | --- | --- | --- | --- | --- | --- | --- | --- |
|  |  | **Levene's Test for Equality of Variances** | | **t-test for Equality of Means** | | | | | | |
|  |  | **F** | **Sig.** | **t** | **df** | **Sig. (2-tailed)** | **Mean Difference** | **Std. Error Difference** | **95% Confidence Interval of the Difference** | |
|  |  |  |  |  |  |  |  |  | **Lower** | **Upper** |
| **BMI** | **Equal variances assumed** | **.28** | .599 | -1.592 | 189 | **.11** | -.91997 | .57795 | -2.06004 | .22009 |
|  | **Equal variances not assumed** |  |  | -1.583 | 181.165 | .115 | -.91997 | .58106 | -2.06649 | .22654 |

**Family History * EE result**

| Crosstab | | | | | |
| --- | --- | --- | --- | --- | --- |
|  |  |  | **EE result** | | **Total** |
|  |  |  | **G1 – Ischemic** | **G2 - Normal** |  |
| **Family History** | **False** | **Count** | 54 | 67 | 121 |
|  |  | **% within EE result** | 58.7% | 66.3% | 62.7% |
|  |  | **% of Total** | 28.0% | 34.7% | 62.7% |
|  | **True** | **Count** | 38 | 34 | 72 |
|  |  | **% within EE result** | **41.3%** | **33.7%** | 37.3% |
|  |  | **% of Total** | 19.7% | 17.6% | 37.3% |
| **Total** | | **Count** | 92 | 101 | 193 |
|  |  | **% within EE result** | 100.0% | 100.0% | 100.0% |
|  |  | **% of Total** | 47.7% | 52.3% | 100.0% |

| Chi-Square Tests | | | | | |
| --- | --- | --- | --- | --- | --- |
|  | **Value** | **df** | **Asymp. Sig. (2-sided)** | **Exact Sig. (2-sided)** | **Exact Sig. (1-sided)** |
| **Pearson Chi-Square** | 1.202(b) | 1 | **.27** |  |  |
| **Continuity Correction(a)** | .897 | 1 | .343 |  |  |
| **Likelihood Ratio** | 1.202 | 1 | .273 |  |  |
| **Fisher's Exact Test** |  |  |  | .299 | .172 |
| **Linear-by-Linear Association** | 1.196 | 1 | .274 |  |  |
| **N of Valid Cases** | 193 |  |  |  |  |

**Alcoolism * EE result**

| Crosstab | | | | | |
| --- | --- | --- | --- | --- | --- |
|  |  |  | **EE result** | | **Total** |
|  |  |  | **G1 - Isquêmico** | **G2 - Normal** |  |
| **alcoolism** | **Do not drink** | **Count** | 52 | 56 | 108 |
|  |  | **% within EE result** | 56.5% | 55.4% | 56.0% |
|  |  | **% of Total** | 26.9% | 29.0% | 56.0% |
|  | **Drink** | **Count** | 40 | 45 | 85 |
|  |  | **% within EE result** | **43.5%** | **44.6%** | 44.0% |
|  |  | **% of Total** | 20.7% | 23.3% | 44.0% |
| **Total** | | **Count** | 92 | 101 | 193 |
|  |  | **% within EE result** | 100.0% | 100.0% | 100.0% |
|  |  | **% of Total** | 47.7% | 52.3% | 100.0% |

| Chi-Square Tests | | | | | |
| --- | --- | --- | --- | --- | --- |
|  | **Value** | **df** | **Asymp. Sig. (2-sided)** | **Exact Sig. (2-sided)** | **Exact Sig. (1-sided)** |
| **Pearson Chi-Square** | .023(b) | 1 | **.88** |  |  |
| **Continuity Correction(a)** | .000 | 1 | .996 |  |  |
| **Likelihood Ratio** | .023 | 1 | .880 |  |  |
| **Fisher's Exact Test** |  |  |  | .886 | .498 |
| **Linear-by-Linear Association** | .023 | 1 | .881 |  |  |
| **N of Valid Cases** | 193 |  |  |  |  |

**Insulin takers * EE result**

| Crosstab | | | | | |
| --- | --- | --- | --- | --- | --- |
|  |  |  | **EE result** | | **Total** |
|  |  |  | **G1 - Ischemic** | **G2 - Normal** |  |
| **Insulin takers** | **False** | **Count** | 60 | 81 | 141 |
|  |  | **% within EE result** | 65.2% | 80.2% | 73.1% |
|  |  | **% of Total** | 31.1% | 42.0% | 73.1% |
|  | **True** | **Count** | 32 | 20 | 52 |
|  |  | **% within EE result** | **34.8%** | **19.8%** | 26.9% |
|  |  | **% of Total** | 16.6% | 10.4% | 26.9% |
| **Total** | | **Count** | 92 | 101 | 193 |
|  |  | **% within EE result** | 100.0% | 100.0% | 100.0% |
|  |  | **% of Total** | 47.7% | 52.3% | 100.0% |

| Chi-Square Tests | | | | | |
| --- | --- | --- | --- | --- | --- |
|  | **Value** | **df** | **Asymp. Sig. (2-sided)** | **Exact Sig. (2-sided)** | **Exact Sig. (1-sided)** |
| **Pearson Chi-Square** | 5.489(b) | 1 | **.02** |  |  |
| **Continuity Correction(a)** | 4.754 | 1 | .029 |  |  |
| **Likelihood Ratio** | 5.514 | 1 | .019 |  |  |
| **Fisher's Exact Test** |  |  |  | .023 | .015 |
| **Linear-by-Linear Association** | 5.461 | 1 | .019 |  |  |
| **N of Valid Cases** | 193 |  |  |  |  |

**Current use of oral hypoglycemiant * EE result**

| Crosstab | | | | | |
| --- | --- | --- | --- | --- | --- |
|  |  |  | **EE result** | | **Total** |
|  |  |  | **G1 - Ischemic** | **G2 - Normal** |  |
| **Current use of**  **oral hypoglycemiant** | **False** | **Count** | 24 | 18 | 42 |
|  |  | **% within EE result** | 26.1% | 17.8% | 21.8% |
|  |  | **% of Total** | 12.4% | 9.3% | 21.8% |
|  | **True** | **Count** | 68 | 83 | 151 |
|  |  | **% within EE result** | **73.9%** | **82.2%** | 78.2% |
|  |  | **% of Total** | 35.2% | 43.0% | 78.2% |
| **Total** | | **Count** | 92 | 101 | 193 |
|  |  | **% within EE result** | 100.0% | 100.0% | 100.0% |
|  |  | **% of Total** | 47.7% | 52.3% | 100.0% |

| Chi-Square Tests | | | | | |
| --- | --- | --- | --- | --- | --- |
|  | **Value** | **df** | **Asymp. Sig. (2-sided)** | **Exact Sig. (2-sided)** | **Exact Sig. (1-sided)** |
| **Pearson Chi-Square** | 1.932(b) | 1 | **.16** |  |  |
| **Continuity Correction(a)** | 1.477 | 1 | .224 |  |  |
| **Likelihood Ratio** | 1.933 | 1 | .164 |  |  |
| **Fisher's Exact Test** |  |  |  | .221 | .112 |
| **Linear-by-Linear Association** | 1.922 | 1 | .166 |  |  |
| **N of Valid Cases** | 193 |  |  |  |  |

**Current use of beta-blockers * EE result**

| Crosstab | | | | | |
| --- | --- | --- | --- | --- | --- |
|  |  |  | **EE result** | | **Total** |
|  |  |  | **G1 - Ischemic** | **G2 - Normal** |  |
| **Current use of**  **beta-blockers** | **False** | **Count** | 47 | 70 | 117 |
|  |  | **% within EE result** | 52.2% | 70.7% | 61.9% |
|  |  | **% of Total** | 24.9% | 37.0% | 61.9% |
|  | **True** | **Count** | 43 | 29 | 72 |
|  |  | **% within EE result** | **47.8%** | **29.3%** | 38.1% |
|  |  | **% of Total** | 22.8% | 15.3% | 38.1% |
| **Total** | | **Count** | 90 | 99 | 189 |
|  |  | **% within EE result** | 100.0% | 100.0% | 100.0% |
|  |  | **% of Total** | 47.6% | 52.4% | 100.0% |

| Chi-Square Tests | | | | | |
| --- | --- | --- | --- | --- | --- |
|  | **Value** | **df** | **Asymp. Sig. (2-sided)** | **Exact Sig. (2-sided)** | **Exact Sig. (1-sided)** |
| **Pearson Chi-Square** | 6.831(b) | 1 | **.009** |  |  |
| **Continuity Correction(a)** | 6.069 | 1 | .014 |  |  |
| **Likelihood Ratio** | 6.862 | 1 | .009 |  |  |
| **Fisher's Exact Test** |  |  |  | .011 | .007 |
| **Linear-by-Linear Association** | 6.794 | 1 | .009 |  |  |
| **N of Valid Cases** | 189 |  |  |  |  |

**Current use of nitrates * EE result**

| Crosstab | | | | | |
| --- | --- | --- | --- | --- | --- |
|  |  |  | **EE result** | | **Total** |
|  |  |  | **G1 - Ischemic** | **G2 - Normal** |  |
| **Current use of**  **nitrates** | **False** | **Count** | 57 | 93 | 150 |
|  |  | **% within EE result** | 63.3% | 93.9% | 79.4% |
|  |  | **% of Total** | 30.2% | 49.2% | 79.4% |
|  | **True** | **Count** | 33 | 6 | 39 |
|  |  | **% within EE result** | **36.7%** | **6.1%** | 20.6% |
|  |  | **% of Total** | 17.5% | 3.2% | 20.6% |
| **Total** | | **Count** | 90 | 99 | 189 |
|  |  | **EE result** | **EE result** | 100.0% | 100.0% |
|  |  | **% of Total** | 47.6% | 52.4% | 100.0% |

| Chi-Square Tests | | | | | |
| --- | --- | --- | --- | --- | --- |
|  | **Value** | **df** | **Asymp. Sig. (2-sided)** | **Exact Sig. (2-sided)** | **Exact Sig. (1-sided)** |
| **Pearson Chi-Square** | 26.965(b) | 1 | **.0000002** |  |  |
| **Continuity Correction(a)** | 25.128 | 1 | .000 |  |  |
| **Likelihood Ratio** | 28.874 | 1 | .000 |  |  |
| **Fisher's Exact Test** |  |  |  | .000 | .000 |
| **Linear-by-Linear Association** | 26.822 | 1 | .000 |  |  |
| **N of Valid Cases** | 189 |  |  |  |  |

**Current use of Calcium channel blockers * EE result**

| Crosstab | | | | | |
| --- | --- | --- | --- | --- | --- |
|  |  |  | **EE result** | | **Total** |
|  |  |  | **G1 - Ischemic** | **G2 - Normal** |  |
| **Current use of**  **Calcium channel**  **blockers** | **False** | **Count** | 57 | 69 | 126 |
|  |  | **% within EE result** | 63.3% | 69.7% | 66.7% |
|  |  | **% of Total** | 30.2% | 36.5% | 66.7% |
|  | **True** | **Count** | 33 | 30 | 63 |
|  |  | **% within EE result** | **36.7%** | **30.3%** | 33.3% |
|  |  | **% of Total** | 17.5% | 15.9% | 33.3% |
| **Total** | | **Count** | 90 | 99 | 189 |
|  |  | **% within EE result** | 100.0% | 100.0% | 100.0% |
|  |  | **% of Total** | 47.6% | 52.4% | 100.0% |

| Chi-Square Tests | | | | | |
| --- | --- | --- | --- | --- | --- |
|  | **Value** | **df** | **Asymp. Sig. (2-sided)** | **Exact Sig. (2-sided)** | **Exact Sig. (1-sided)** |
| **Pearson Chi-Square** | .859(b) | 1 | **.35** |  |  |
| **Continuity Correction(a)** | .597 | 1 | .440 |  |  |
| **Likelihood Ratio** | .859 | 1 | .354 |  |  |
| **Fisher's Exact Test** |  |  |  | .360 | .220 |
| **Linear-by-Linear Association** | .855 | 1 | .355 |  |  |
| **N of Valid Cases** | 189 |  |  |  |  |

**Failure to Achieve 85% of the Maximal Age-predicted Heart Rate * EE result**

| Crosstab | | | | | |
| --- | --- | --- | --- | --- | --- |
|  |  |  | **EE result** | | **Total** |
|  |  |  | **G1 – Ischemic** | **G2 - Normal** |  |
| **Failure to Achieve 85% of the Maximal Age-predicted Heart Rate** | **false** | **Count** | 62 | 70 | 132 |
|  |  | **% within EE result** | 67.4% | 69.3% | 68.4% |
|  |  | **% of Total** | 32.1% | 36.3% | 68.4% |
|  | **True** | **Count** | 30 | 31 | 61 |
|  |  | **% within EE result** | **32.6%** | **30.7%** | 31.6% |
|  |  | **% of Total** | 15.5% | 16.1% | 31.6% |
| **Total** | | **Count** | 92 | 101 | 193 |
|  |  | **% within EE result** | 100.0% | 100.0% | 100.0% |
|  |  | **% of Total** | 47.7% | 52.3% | 100.0% |

| Chi-Square Tests | | | | | |
| --- | --- | --- | --- | --- | --- |
|  | **Value** | **df** | **Asymp. Sig. (2-sided)** | **Exact Sig. (2-sided)** | **Exact Sig. (1-sided)** |
| **Pearson Chi-Square** | .082(b) | 1 | **.77** |  |  |
| **Continuity Correction(a)** | .017 | 1 | .896 |  |  |
| **Likelihood Ratio** | .082 | 1 | .775 |  |  |
| **Fisher's Exact Test** |  |  |  | .877 | .448 |
| **Linear-by-Linear Association** | .081 | 1 | .776 |  |  |
| **N of Valid Cases** | 193 |  |  |  |  |

**Achieved 85% of the Maximal Age-predicted Heart Rate * EE result**

| Crosstab | | | | | |
| --- | --- | --- | --- | --- | --- |
|  |  |  | **EE result** | | **Total** |
|  |  |  | **G1 - Ischemic** | **G2 - Normal** |  |
| **Achieved 85% of the Maximal Age-predicted Heart Rate** | **False** | **Count** | 60 | 77 | 137 |
|  |  | **% within EE result** | 65.2% | 76.2% | 71.0% |
|  |  | **% of Total** | 31.1% | 39.9% | 71.0% |
|  | **True** | **Count** | 32 | 24 | 56 |
|  |  | **% within EE result** | **34.8%** | **23.8%** | 29.0% |
|  |  | **% of Total** | 16.6% | 12.4% | 29.0% |
| **Total** | | **Count** | 92 | 101 | 193 |
|  |  | **% within EE result** | 100.0% | 100.0% | 100.0% |
|  |  | **% of Total** | 47.7% | 52.3% | 100.0% |

| Chi-Square Tests | | | | | |
| --- | --- | --- | --- | --- | --- |
|  | **Value** | **df** | **Asymp. Sig. (2-sided)** | **Exact Sig. (2-sided)** | **Exact Sig. (1-sided)** |
| **Pearson Chi-Square** | 2.839(b) | 1 | **.092** |  |  |
| **Continuity Correction(a)** | 2.329 | 1 | .127 |  |  |
| **Likelihood Ratio** | 2.842 | 1 | .092 |  |  |
| **Fisher's Exact Test** |  |  |  | .113 | .063 |
| **Linear-by-Linear Association** | 2.824 | 1 | .093 |  |  |
| **N of Valid Cases** | 193 |  |  |  |  |

**Achieved Maximal Age-predicted Heart Rate * EE result**

| Crosstab | | | | | |
| --- | --- | --- | --- | --- | --- |
|  |  |  | **EE result** | | **Total** |
|  |  |  | **G1 – Ischemic** | **G2 - Normal** |  |
| **Achieved Maximal**  **Age-predicted Heart Rate** | **False** | **Count** | 80 | 78 | 158 |
|  |  | **% within EE result** | 87.0% | 77.2% | 81.9% |
|  |  | **% of Total** | 41.5% | 40.4% | 81.9% |
|  | **True** | **Count** | 12 | 23 | 35 |
|  |  | **% within EE result** | **13.0%** | **22.8%** | 18.1% |
|  |  | **% of Total** | 6.2% | 11.9% | 18.1% |
| **Total** | | **Count** | 92 | 101 | 193 |
|  |  | **% within EE result** | 100.0% | 100.0% | 100.0% |
|  |  | **% of Total** | 47.7% | 52.3% | 100.0% |

| Chi-Square Tests | | | | | |
| --- | --- | --- | --- | --- | --- |
|  | **Value** | **df** | **Asymp. Sig. (2-sided)** | **Exact Sig. (2-sided)** | **Exact Sig. (1-sided)** |
| **Pearson Chi-Square** | 3.069(b) | 1 | **.08** |  |  |
| **Continuity Correction(a)** | 2.449 | 1 | .118 |  |  |
| **Likelihood Ratio** | 3.122 | 1 | .077 |  |  |
| **Fisher's Exact Test** |  |  |  | .094 | .058 |
| **Linear-by-Linear Association** | 3.054 | 1 | .081 |  |  |
| **N of Valid Cases** | 193 |  |  |  |  |

**Achieved Above Maximal Age-predicted Heart Rate * EE result**

| Crosstab | | | | | |
| --- | --- | --- | --- | --- | --- |
|  |  |  | **EE result** | | **Total** |
|  |  |  | **G1 - Ischemic** | **G2 - Normal** |  |
| **Achieved Above Maximal Age-predicted Heart Rate** | **False** | **Count** | 37 | 33 | 70 |
|  |  | **% within EE result** | 92.5% | 67.3% | 78.7% |
|  |  | **% of Total** | 41.6% | 37.1% | 78.7% |
|  | **True** | **Count** | 3 | 16 | 19 |
|  |  | **% within EE result** | **7.5%** | **32.7%** | 21.3% |
|  |  | **% of Total** | 3.4% | 18.0% | 21.3% |
| **Total** | | **Count** | 40 | 49 | 89 |
|  |  | **% within EE result** | 100.0% | 100.0% | 100.0% |
|  |  | **% of Total** | 44.9% | 55.1% | 100.0% |

| Chi-Square Tests | | | | | |
| --- | --- | --- | --- | --- | --- |
|  | **Value** | **df** | **Asymp. Sig. (2-sided)** | **Exact Sig. (2-sided)** | **Exact Sig. (1-sided)** |
| **Pearson Chi-Square** | 8.298(b) | 1 | **.004** |  |  |
| **Continuity Correction(a)** | 6.868 | 1 | .009 |  |  |
| **Likelihood Ratio** | 9.082 | 1 | .003 |  |  |
| **Fisher's Exact Test** |  |  |  | .004 | .003 |
| **Linear-by-Linear Association** | 8.205 | 1 | .004 |  |  |
| **N of Valid Cases** | 89 |  |  |  |  |

| Group Statistics | | | | | |
| --- | --- | --- | --- | --- | --- |
|  | **EE result** | **N** | **Mean** | **Std. Deviation** | **Std. Error Mean** |
| **Ejection fraction** | **G1 - Ischemic** | 90 | **.639** | **.075** | .00793 |
|  | **G2 – Normal** | 100 | **.664** | **.052** | .00522 |
| **Resting heart rate** | **G1 - Ischemic** | 90 | **113.5** | **35.9** | 3.785 |
|  | **G2 – Normal** | 101 | **116.2** | **39.3** | 3.906 |
| **Peak exercise heart rate** | **G1 - Ischemic** | 90 | **164.0** | **29.2** | 3.08213 |
|  | **G2 – Normal** | 101 | **170.5** | **25.6** | 2.54277 |
| **Final heart rate** | **G1 – Ischemic** | 90 | **110.2** | **27.6** | 2.91072 |
|  | **G2 – Normal** | 101 | **114.4** | **26.8** | 2.66440 |
| **Resting Systolic Blood Pressure** | **G1 – Ischemic** | 90 | **159.6** | **30.4** | 3.20724 |
|  | **G2 – Normal** | 101 | **163.8** | **36.3** | 3.61664 |
| **Resting Diastolic Blood Pressure** | **G1 – Ischemic** | 90 | **86.7** | **18.6** | 1.96569 |
|  | **G2 – Normal** | 101 | **83.1** | **14.7** | 1.46215 |
| **Peak exercise Systolic Blood Pressure** | **G1 – Ischemic** | 89 | **162.2** | **32.9** | 3.48277 |
|  | **G2 – Normal** | 101 | **163.0** | **31.2** | 3.10445 |
| **Peak exercise Diastolic Blood Pressure** | **G1 – Ischemic** | 89 | **88.4** | **11.6** | 1.23418 |
|  | **G2 – Normal** | 101 | **87.5** | **9.8** | .97398 |
| **Final Systolic Blood Pressure** | **G1 – Ischemic** | 90 | **107.6** | **35.2** | 3.71104 |
|  | **G2 – Normal** | 101 | **108.8** | **36.0** | 3.58224 |
| **Final Diastolic Blood Pressure** | **G1 - Ischemic** | 90 | **35.8** | **40.3** | 4.24405 |
|  | **G2 - Normal** | 101 | **38.1** | **38.6** | 3.83938 |

| Independent Samples Test | | | | | | | | | | |
| --- | --- | --- | --- | --- | --- | --- | --- | --- | --- | --- |
|  |  | **Levene's Test for Equality of Variances** | | **t-test for Equality of Means** | | | | | | |
|  |  | **F** | **Sig.** | **t** | **df** | **Sig. (2-tailed)** | **Mean Difference** | **Std. Error Difference** | **95% Confidence Interval of the Difference** | |
|  |  |  |  |  |  |  |  |  | **Lower** | **Upper** |
| **Ejection fraction** | **Equal variances assumed** | 6.772 | **.01** | -2.728 | 188 | .007 | -.02541 | .00932 | -.04379 | -.00704 |
|  | **Equal variances not assumed** |  |  | -2.678 | 156.363 | **.008** | -.02541 | .00949 | -.04416 | -.00667 |
| **Resting heart rate** | **Equal variances assumed** | 2.228 | .137 | -.490 | 189 | **.62** | -2.679 | 5.468 | -13.465 | 8.106 |
|  | **Equal variances not assumed** |  |  | -.493 | 188.864 | .623 | -2.679 | 5.439 | -13.409 | 8.051 |
| **Peak exercise heart rate** | **Equal variances assumed** | 3.304 | .071 | -1.628 | 189 | **.11** | -6.45424 | 3.96478 | -14.27515 | 1.36668 |
|  | **Equal variances not assumed** |  |  | -1.615 | 177.995 | .108 | -6.45424 | 3.99564 | -14.33916 | 1.43069 |
| **Final heart rate** | **Equal variances assumed** | .493 | .484 | -1.071 | 189 | **.29** | -4.21826 | 3.93903 | -11.98838 | 3.55185 |
|  | **Equal variances not assumed** |  |  | -1.069 | 185.021 | .286 | -4.21826 | 3.94605 | -12.00330 | 3.56678 |
| **Resting Systolic Blood Pressure** | **Equal variances assumed** | 9.194 | **.003** | -.866 | 189 | .39 | -4.22662 | 4.88338 | -13.85956 | 5.40632 |
|  | **Equal variances not assumed** |  |  | -.874 | 188.289 | **.38** | -4.22662 | 4.83389 | -13.76217 | 5.30893 |
| **Resting diastolic blood pressure** | **Equal variances assumed** | .766 | .383 | 1.468 | 189 | .144 | 3.54785 | 2.41690 | -1.21972 | 8.31543 |
|  | **Equal variances not assumed** |  |  | 1.448 | 168.754 | .149 | 3.54785 | 2.44986 | -1.28846 | 8.38417 |
| **Peak exercise systolic blood pressure** | **Equal variances assumed** | .140 | .709 | -.174 | 188 | .862 | -.80899 | 4.65025 | -9.98237 | 8.36439 |
|  | **Equal variances not assumed** |  |  | -.173 | 182.183 | .863 | -.80899 | 4.66555 | -10.01444 | 8.39646 |
| **Peak exercise diastolic blood pressure** | **Equal variances assumed** | .216 | .643 | .580 | 188 | .563 | .90221 | 1.55515 | -2.16557 | 3.97000 |
|  | **Equal variances not assumed** |  |  | .574 | 172.772 | .567 | .90221 | 1.57221 | -2.20100 | 4.00542 |
| **Final systolic blood pressure** | **Equal variances assumed** | .314 | .576 | -.243 | 189 | .808 | -1.25633 | 5.16460 | -11.44399 | 8.93134 |
|  | **Equal variances not assumed** |  |  | -.244 | 187.356 | .808 | -1.25633 | 5.15793 | -11.43141 | 8.91875 |
| **Final diastolic blood pressure** | **Equal variances assumed** | .577 | .448 | -.410 | 189 | .682 | -2.34103 | 5.70893 | -13.60244 | 8.92038 |
|  | **Equal variances not assumed** |  |  | -.409 | 184.377 | .683 | -2.34103 | 5.72300 | -13.63203 | 8.94996 |

| Descriptive Statistics | | | | |
| --- | --- | --- | --- | --- |
|  | **N** | **Percentiles** | | |
|  |  | **25th** | **50th (Median)** | **75th** |
| **Resting Wall Motion Score Index of the left ventricle** | 191 | 1,0000 | 1,0000 | 1,0600 |
| **Peak Exercise Wall Motion Score Index of the left ventricle** | 192 | 1,0000 | 1,0000 | 1,1200 |
| **EE Result** | 193 | 1,0000 | 1,0000 | 2,0000 |

Mann-Whitney Test

| Ranks | | | | |
| --- | --- | --- | --- | --- |
|  | **EE result** | **N** | **Mean Rank** | **Sum of Ranks** |
| **Resting Wall Motion Score Index of the left ventricle** | **Normal** | 99 | 68,00 | 6732,00 |
|  | **Ischemic** | 92 | 126,13 | 11604,00 |
|  | **Total** | 191 |  |  |
| **Peak exercise Wall Motion Score Index of the left ventricle** | **Normal** | 100 | 51,12 | 5112,00 |
|  | **Ischemic** | 92 | 145,83 | 13416,00 |
|  | **Total** | 192 |  |  |

| Test Statistics(a) | | |
| --- | --- | --- |
|  | **Resting Wall Motion Score Index of the left ventricle** | **Peak exercise Wall Motion Score Index of the left ventricle** |
| **Mann-Whitney U** | 1782,000 | 62,000 |
| **Wilcoxon W** | 6732,000 | 5112,000 |
| **Z** | -9,039 | -12,772 |
| **Asymp. Sig. (2-tailed)** | ,000 | ,000 |
| a Grouping Variable: EE result | | |

**Event: Myocardial infarction * EE result**

| Crosstab | | | | | |
| --- | --- | --- | --- | --- | --- |
|  |  |  | **EE result** | | **Total** |
|  |  |  | **G1 - Isquêmico** | **G2 - Normal** |  |
| **Event: myocardial**  **infarction** | **False** | **Count** | 88 | 99 | 187 |
|  |  | **% within EE result** | 95.7% | 98.0% | 96.9% |
|  |  | **% of Total** | 45.6% | 51.3% | 96.9% |
|  | **True** | **Count** | 4 | 2 | 6 |
|  |  | **% within EE result** | **4.3%** | **2.0%** | 3.1% |
|  |  | **% of Total** | 2.1% | 1.0% | 3.1% |
| **Total** | | **Count** | 92 | 101 | 193 |
|  |  | **% within EE result** | 100.0% | 100.0% | 100.0% |
|  |  | **% of Total** | 47.7% | 52.3% | 100.0% |

| Chi-Square Tests | | | | | | |
| --- | --- | --- | --- | --- | --- | --- |
|  | **Value** | **df** | **Asymp. Sig. (2-sided)** | **Exact Sig. (2-sided)** | **Exact Sig. (1-sided)** | **Point Probability** |
| **Pearson Chi-Square** | .896(b) | 1 | .344 | **.43** | .298 |  |
| **Continuity Correction(a)** | .282 | 1 | .595 |  |  |  |
| **Likelihood Ratio** | .907 | 1 | .341 | .427 | .298 |  |
| **Fisher's Exact Test** |  |  |  | .427 | .298 |  |
| **Linear-by-Linear Association** | .891(c) | 1 | .345 | .427 | .298 | .213 |
| **N of Valid Cases** | 193 |  |  |  |  |  |

**Cardiac death * EE result**

| Crosstab | | | | | |
| --- | --- | --- | --- | --- | --- |
|  |  |  | **EE result** | | **Total** |
|  |  |  | **G1 - Ischemic** | **G2 - Normal** |  |
| **Cardiac death** | **False** | **Count** | 88 | 99 | 187 |
|  |  | **% within EE result** | 95.7% | 98.0% | 96.9% |
|  |  | **% of Total** | 45.6% | 51.3% | 96.9% |
|  | **True** | **Count** | 4 | 2 | 6 |
|  |  | **% within EE result** | **4.3%** | **2.0%** | 3.1% |
|  |  | **% of Total** | 2.1% | 1.0% | 3.1% |
| **Total** | | **Count** | 92 | 101 | 193 |
|  |  | **% within EE result** | 100.0% | 100.0% | 100.0% |
|  |  | **% of Total** | 47.7% | 52.3% | 100.0% |

| Chi-Square Tests | | | | | | |
| --- | --- | --- | --- | --- | --- | --- |
|  | **Value** | **df** | **Asymp. Sig. (2-sided)** | **Exact Sig. (2-sided)** | **Exact Sig. (1-sided)** | **Point Probability** |
| **Pearson Chi-Square** | .896(b) | 1 | .344 | **.43** | .298 |  |
| **Continuity Correction(a)** | .282 | 1 | .595 |  |  |  |
| **Likelihood Ratio** | .907 | 1 | .341 | .427 | .298 |  |
| **Fisher's Exact Test** |  |  |  | .427 | .298 |  |
| **Linear-by-Linear Association** | .891(c) | 1 | .345 | .427 | .298 | .213 |
| **N of Valid Cases** | 193 |  |  |  |  |  |

**Event: Surgical myocardial revascularization * EE result**

| Crosstab | | | | | |
| --- | --- | --- | --- | --- | --- |
|  |  |  | **EE result** | | **Total** |
|  |  |  | **G1 – Ischemic** | **G2 - Normal** |  |
| **Event: Surgical myocardial revascularization** | **Falso** | **Count** | 86 | 100 | 186 |
|  |  | **% within EE result** | 93.5% | 99.0% | 96.4% |
|  |  | **% of Total** | 44.6% | 51.8% | 96.4% |
|  | **Verdadeiro** | **Count** | 6 | 1 | 7 |
|  |  | **% within EE result** | **6.5%** | **1.0%** | 3.6% |
|  |  | **% of Total** | 3.1% | .5% | 3.6% |
| **Total** | | **Count** | 92 | 101 | 193 |
|  |  | **% within EE result** | 100.0% | 100.0% | 100.0% |
|  |  | **% of Total** | 47.7% | 52.3% | 100.0% |

| Chi-Square Tests | | | | | | |
| --- | --- | --- | --- | --- | --- | --- |
|  | **Value** | **df** | **Asymp. Sig. (2-sided)** | **Exact Sig. (2-sided)** | **Exact Sig. (1-sided)** | **Point Probability** |
| **Pearson Chi-Square** | 4.215(b) | 1 | .040 | **.06** | .046 |  |
| **Continuity Correction(a)** | 2.781 | 1 | .095 |  |  |  |
| **Likelihood Ratio** | 4.597 | 1 | .032 | .055 | .046 |  |
| **Fisher's Exact Test** |  |  |  | .055 | .046 |  |
| **Linear-by-Linear Association** | 4.193(c) | 1 | .041 | .055 | .046 | .041 |
| **N of Valid Cases** | 193 |  |  |  |  |  |

**Event: Percutaneous myocardial revascularization * EE result**

| Crosstab | | | | | |
| --- | --- | --- | --- | --- | --- |
|  |  |  | **EE result** | | **Total** |
|  |  |  | **G1 - Ischemic** | **G2 - Normal** |  |
| **Event: Percutaneous myocardial revascularization** | **False** | **Count** | 87 | 99 | 186 |
|  |  | **% within EE result** | 94.6% | 98.0% | 96.4% |
|  |  | **% of Total** | 45.1% | 51.3% | 96.4% |
|  | **True** | **Count** | 5 | 2 | 7 |
|  |  | **% within EE result** | 5.4% | 2.0% | 3.6% |
|  |  | **% of Total** | 2.6% | 1.0% | 3.6% |
| **Total** | | **Count** | 92 | 101 | 193 |
|  |  | **% within EE result** | 100.0% | 100.0% | 100.0% |
|  |  | **% of Total** | 47.7% | 52.3% | 100.0% |

| Chi-Square Tests | | | | | | |
| --- | --- | --- | --- | --- | --- | --- |
|  | **Value** | **df** | **Asymp. Sig. (2-sided)** | **Exact Sig. (2-sided)** | **Exact Sig. (1-sided)** | **Point Probability** |
| **Pearson Chi-Square** | 1.644(b) | 1 | .200 | .261 | .186 |  |
| **Continuity Correction(a)** | .804 | 1 | .370 |  |  |  |
| **Likelihood Ratio** | 1.683 | 1 | .195 | .261 | .186 |  |
| **Fisher's Exact Test** |  |  |  | .261 | .186 |  |
| **Linear-by-Linear Association** | 1.635(c) | 1 | .201 | .261 | .186 | .140 |
| **N of Valid Cases** | 193 |  |  |  |  |  |
